# Convergent Evolution in Amblyopsid Cavefishes and the Age of Eastern North American Subterranean Ecosystems

**DOI:** 10.1093/molbev/msaf185

**Published:** 2025-08-05

**Authors:** Chase D Brownstein, Maxime Policarpo, Richard C Harrington, Eva A Hoffman, Maya F Stokes, Didier Casane, Thomas J Near

**Affiliations:** Department of Ecology and Evolutionary Biology, Yale University, New Haven, CT, USA; Zoological Institute, Department of Environmental Sciences, University of Basel, Basel, Switzerland; Evolution of Sensory and Physiological Systems, Max Planck Institute for Biological Intelligence, Martinsried, Germany; South Carolina Department of Natural Resources, Marine Resources Institute, Charleston, SC, USA; Division of Paleontology, American Museum of Natural History, New York, NY, USA; Department of Earth, Ocean, & Atmospheric Science, Florida State University, Tallahassee, FL, USA; CNRS, IRD, UMR Évolution, Génomes, Comportement et Écologie, Université Paris-Saclay, Gif-sur-Yvette 91198, France; UFR Sciences du Vivant, Université Paris Cité, Paris 75013, France; Department of Ecology and Evolutionary Biology, Yale University, New Haven, CT, USA; Yale Peabody Museum, New Haven, CT, USA

**Keywords:** cave, phylogenetics, genomics, geochronology, geogenomics, convergent evolution, relaxed selection, biological sciences, evolution

## Abstract

Genomes provide tools for reconstructing organismal evolution and larger Earth system processes. Although genome sequences have been jointly analyzed with geological data to understand links between biological evolution and geological phenomena such as erosion and uplift, genomic and natural history observations have seldom been leveraged to reconstruct the timescale of landscape change in cases where traditional methods from the Earth sciences cannot. Here, we reconstruct the genomic evolution of cave-adapted amblyopsid fishes. Although high-resolution computed tomography reveals the strikingly similar skeletons of cave-adapted lineages, our analyses of the genomes of all species in this clade suggest that amblyopsids independently colonized caves and degenerated their eyes at least four times after descending from populations that already possessed adaptations to low-light environments. By examining pseudogenization through loss-of-function mutations in amblyopsids, we infer that the genomic bases of their vision degenerated over millions of years. We leverage these data to infer the ages of subterranean karstic ecosystems in eastern North America, which are difficult to date using standard geochronologic techniques. Our results support ancient ages for imperiled North American cave biotas and show how genomes can be used to inform the timescale of landscape evolution.

## Introduction

The origins of adaptations for life in new environments have captivated evolutionary biologists since the discipline began (Darwin 1859). One of the most striking discoveries has been that lineages across the Tree of Life have convergently modified their phenotypes via strikingly similar genetic and developmental trajectories (Hoekstra 2006; Manceau et al. 2010; Rosenblum et al. 2010; Losos 2011; Stern 2013; Gallant et al. 2014; Agrawal 2017).

Caves are a classic system for studying the convergent degeneration of traits under changing selective landscapes. Since the 19th century, thousands of obligate cave-dwelling animals have been described (Rétaux and Casane 2013; Culver and Pipan 2019; Niemiller and Taylor 2019). These dark, nutrient-limited environments (Poulson and White 1969) often necessitate the evolution of novel features and facilitate the degeneration of traits suited for life at the surface due to the relaxation of selective pressures or selection against metabolically costly structures that are of less use in caves (e.g. eyes). Degenerative evolution has often been examined in cave-adapted model organisms, especially blind populations of *Astyanax mexicanus* fish (Jeffery et al. 2003; Rétaux and Casane 2013; McGaugh et al. 2014; Moran et al. 2015; Casane and Rétaux 2016; Krishnan and Rohner 2017; Gore et al. 2018). However, the macroevolution of life in caves remains obscure (Niemiller et al. 2013a; Yang et al. 2016; Hart et al. 2020; Mao et al. 2021, 2022; Balart-García et al. 2023).

One essential aspect of cave ecosystems that has often evaded resolution are the ages of the landforms themselves. The species-rich subterranean ecosystems of eastern North America, for example, are notoriously challenging to date via traditional geochronologic techniques (Granger et al. 2001; Osborne 2005, 2007; Stock et al. 2005 ; White 2007a). An emerging field, geogenomics, seeks to test hypotheses about landform evolution by studying the history of biological diversification recorded in genomic data (Baker et al. 2014; Dawson et al. 2022; Dolby et al. 2022). Yet, phylogenetic analysis of genome-scale data has not yet been leveraged to investigate the history of cave system formation.

Here, we reconstruct the evolutionary history of cavefishes in the clade *Amblyopsidae*, which is part of an ancient, species-poor lineage of fishes (*Percopsiformes*) endemic to North America (Niemiller and Poulson 2010, Niemiller et al. 2012, 2013a,b; Armbruster et al. 2016; Hart et al. 2020, 2025). Amblyopsid cavefishes include facultative and obligate cave dwellers that show classic adaptations for subterranean life, such as the expansion of sensory structures on the head and body, as well as the degeneration of numerous traits associated with life at the surface, including loss of pigmentation, the degeneration of the eye, and the reduction of hearing capabilities (Niemiller and Poulson 2010; Niemiller et al. 2013a,c; Hart et al. 2020). By using genome sequences to produce a new hypothesis of amblyopsid relationships, we demonstrate that at least four lineages with similar skeletal adaptations show genomic evidence for independent cave colonization. Our inferences of cavefish genomic evolution suggest multiple ancient invasions of cave environments by amblyopsids that can be used to inform the ages of the biodiverse subterranean ecosystems of eastern North America (Niemiller and Taylor 2019; Niemiller et al. 2021).

## Materials and Methods

### High-Resolution Computed Tomography

In order to investigate and characterize the osteology of cavefishes, we conducted high resolution computed tomography (CT) scanning on multiple individuals for each of the 12 species of *Percopsiformes* recognized in this study except for *Troglichthys rosae*, for which we obtained CT scans from the Tulane University Biodiversity Research Institute, Royal D. Suttkus Fish Collections, via Morphosource.org (TU Fish 22675). All CT scans that we present in this study were scanned with a Nikon XT H 225 ST system at Yale University. Scan parameters and specimens CT scans are in supplementary table S1, Supplementary Material online. Raw CT image stacks were segmented and rendered using 3DSlicer (Kikinis et al. 2014) and Blender (blender.org). We refer the readers to supplementary figs. S1 to S3, Supplementary Material online for plates comparing the cranial (supplementary fig. S1, Supplementary Material online) and postcranial (supplementary fig. S2, Supplementary Material online) anatomy of different percopsiform species and highlighting key character state changes (supplementary fig. S3, Supplementary Material online).

### Bayesian Analysis of the Morphological Dataset

In order to understand the phylogenetic relationships of extinct taxa potentially useful as node and tip calibrations, we conducted a Bayesian analysis using the morphological matrix presented in a recent study (Murray et al. 2020), which includes 14 extinct and 7 extant operational taxonomic units. We provide the following justifications for the ages of extinct taxa included in our dataset:

†*Cumbaaichthys oxyrhynchus*
**Stratigraphy**: Lacdes Bois, Northwest Territories, Canada: early Turonian, Late Cretaceous (Murray 2014).
**Fossil tip age**: 93.9 Ma (Wilson 1977; Mayr et al. 2019).
†*Boreiohydrias dayi*
**Stratigraphy**: Lacdes Bois, Northwest Territories, Canada: early Turonian, Late Cretaceous (Murray 2014).
**Fossil tip age**: 93.9 Ma.
†*Sphenocephalus* spp.
**Stratigraphy**: Baumberg and Sendenhorst, Westphalia, Germany: late Campanian, Late Cretaceous (Newbrey et al. 2013).
**Fossil tip age**: 72.1 Ma.
†*Xenyllion zonensis*
**Stratigraphy**: Fish Scales Formation, Alberta, Canada: early Cenomanian, Late Cretaceous (Newbrey et al. 2013)
**Fossil tip age**: 97.2 Ma.
†*Xenyllion stewarti*
**Stratigraphy**: Mowry Shale Formation, Vernal, Utah, USA: earliest Cenomanian, Late Cretaceous (Newbrey et al. 2013).
**Fossil tip age**: 100.5 Ma (Gradstein et al. 2021).
†*Libotonius pearsoni*
**Stratigraphy**: Klondike Mountain Formation, Okanogan County, Washington, USA: Middle Eocene (Wilson 1979).
**Fossil tip age**: 51.2 Ma (Rubino et al. 2021).
†*Libotonius blakeburnensis*
**Stratigraphy**: Whipsaw Creek, Allenby Formation, British Columbia, Canada: Ypresian, Eocene (Wilson 1977), between 56 and 48.1 Ma (Near and Thacker 2024).
**Fossil tip age**: 52.05 Ma
†*Mcconichthys longipinnis*
**Stratigraphy**: Bonin Schoolhouse, Tullock Member, Fort Union Formation, McCone County, Montana, USA (Grande 1988): Danian, 66.02 to 61.7 Ma (Near and Thacker 2024)
**Fossil tip age**: 63.85 Ma.
†*Amphiplaga brachyptera*
**Stratigraphy**: Fossil Lake Sample Site H-1 [Thompson Ranch Quarry; Locality H], Fossil Butte Member, Green River Formation, Lincoln County, Wyoming, USA: Ypresian, 56.0 to 48.1 Ma (Near and Thacker 2024).
**Fossil tip age**: 52.05 Ma.
†*Erismatopterus levatus*
**Stratigraphy**: Lake Gosiute and Lake Uinta deposits, Green River Formation, USA: Ypresian, 56.0 to 48.1 Ma (Near and Thacker 2024).
**Fossil tip age**: 52.05 Ma.
†*Massamorichthys wilsoni*
**Stratigraphy**: Joffre Bridge road cut, Paskapoo Formation, Alberta, Canada: Selandian to Thanetian, Paleocene, 61.7 to 56.0 Ma (Murray 1996; Near and Thacker 2024).
**Fossil tip age**: 58.85 Ma.
†*Lateopisciculus turrifumosus*
**Stratigraphy**: Joffre Bridge road cut, Paskapoo Formation, Alberta, Canada: Selandian to Thanetian, Paleocene, 61.7 to 56.0 Ma (Murray and Wilson 1996; Near and Thacker 2024).
**Fossil tip age**: 58.85 Ma.
†*Trichophanes foliarum*
**Stratigraphy**: Florissant Fossil Beds, Colorado, USA: Priabonian, Eocene 37.7 to 33.9 Ma (Near and Thacker 2024).
**Fossil tip age**: 35.8 Ma.
†*Lindoeichthys albertensis*
**Stratigraphy**: Pisces Point locality, Scollard Formation, Dry Island Buffalo Jump Provincial Park, Alberta, Canada: early middle Maastrichtian, Late Cretaceous (Murray et al. 2020), 72.1 to 66.02 Ma (Gradstein et al. 2021).
**Fossil tip age**: 69.1 Ma.

We imported the morphological matrix into the BEAUTi terminal to build an xml file for analysis in BEAST v 2.6.7 (Bouckaert et al. 2014; Bouckaert et al. 2019). We partitioned characters by state and used the Mk_v_ model of character evolution (Lewis 2001). We ran the analysis using the BEAST implementation of the Fossilized Birth Death Model (Gavryushkina et al. 2016) and set the rho parameter to one since all major living percopsiform clades are sampled in the matrix. We used a lognormal relaxed clock as the clock prior and we set the root origin time to 122.0 Ma, the age of †*Acanthomorphum* otoliths representing potentially the oldest definite occurrences of crown *Acanthomorpha* in the fossil record (Chen et al. 2014), with bounds of 100.5 Ma (the Aptian-Albian boundary, representing the ages of the oldest known representatives of ingroup acanthomorph clades; see Chen et al. 2014; Davesne et al. 2016; Delbarre et al. 2016; Brownstein and Near 2024) and 145 Ma (the Jurassic-Cretaceous boundary, and the approximate age of the acanthomorph crown in recent molecular phylogenies) (Alfaro et al. 2018; Ghezelayagh et al. 2022). Next, we set the diversification rate prior to 0.05, the background rate in *Acanthomorpha* (Ghezelayagh et al. 2022), with bounds of 1.0 and 0.0. We ran two independent Markov Chain Monte Carlo chains over 5.0 × 10^7^ generations with a 1.0 × 10^6^ preburnin, assessed for convergence of the posteriors and high ESS values using Tracer v. 1.7 (Rambaut et al. 2018), and combined and summarized the posterior tree sets in a single maximum clade credibility tree with median node heights using LogCombiner v. 2.6.6 and TreeAnnotator v 2.6.6 (Bouckaert et al. 2014; Bouckaert et al. 2019). The resulting time tree is shown in supplementary fig. S4, Supplementary Material online.

### UCE Dataset Assembly

Our dataset includes UCE sequence data from previously published UCE-target-enriched studies (Alfaro et al. 2018; Hart et al. 2020; Ghezelayagh et al. 2022) and UCE data harvested from published whole genome sequences (Malmstrøm et al. 2016; Hart et al. 2025; Niemiller et al. 2025). NCBI SRA BioProject and BioSample accession numbers are provided in supplementary table S2, Supplementary Material online. For UCE-target-enriched data, we removed adapter sequences from demultiplexed raw read files using illumiprocessor (Anon) and Trimmomatic (Bolger et al. 2014) and generated assemblies using SPAdes (Bankevich et al. 2012). To extract UCE sequence data from whole genomes we downloaded genomes from NCBI and converted to 2bit format using faToTwoBit from the UCSC Genome Browser Downloads (https://hgdownload.cse.ucsc.edu/admin/exe/). We generated and used an SQLite database to identify UCE loci in each genome assembly by running the PHYLUCE v.1.7.1 program run_multiple_lastzs_sqlite.py (Faircloth 2016, 2024), using default parameters and the FASTA file containing the UCE bait sequences. We extracted the identified UCE loci along with ±500 bp of sequence from each flank using phyluce_probe_slice_sequence_from_genomes.py with default parameters. We generated alignments with MAFFT v. 7.475 (Katoh and Standley 2013) and trimmed them using Gblocks v0.91 (Castresana 2000).

### Phylogenetic Analysis of the UCE Dataset

We analyzed our UCE dataset using the maximum likelihood program IQ-TREE v 2.2.0 (Minh et al. 2020b) on the Yale McCleary High Performance Computing Cluster. We ran analyses on a concatenated version of the 75% complete UCE dataset in which we either treated whole concatenated sequences as single partitions or used PartitionFinder2 to select a best-fit partitioning scheme (Lanfear et al. 2016). In both analyses of the concatenated dataset, we assessed nodal support using 1,000 ultrafast bootstraps. We also inferred gene trees for individual UCEs using optimal models of nucleotide evolution found using ModelFinderPlus (Kalyaanamoorthy et al. 2017) and then summarized them in a single species tree using the coalescent model implemented in ASTRAL-III (Zhang et al. 2018). Finally, we calculated gene and site concordance factors using 100 subsampled quartets from the concatenated alignment to provide an additional metric of support for inferred topologies (Minh et al. 2020a). These trees are shown in supplementary fig. S5, Supplementary Material online, and concordance factors are shown in supplementary fig. S6, Supplementary Material online.

Next, we used the Yale McCleary High Performance Computing Cluster to conduct Bayesian tip and node-dating analyses on three sets of 50 UCE alignments randomly selected from our UCE dataset to produce a hypothesis of the timescale of evolution of *Percopsiformes* and assess how calibration strategy influences our inference of divergence times in this lineage. For these analyses, we used a GTR model of nucleotide evolution, a lognormal relaxed clock, and the BEAST implementation of the FBD model (Gavryushkina et al. 2016). For the tip-dating analyses, we fixed the position of fossil calibrations, inputted as “NNN” strings in the alignment file, using monophyletic MRCA priors along the branches in *Percopsiformes* where they were placed in our tip-dated analysis of the morphological dataset. In the node-dating analysis, we used a subset of the fossil calibrations to fix a prior to the MRCA of *Aphredoderus* and *Amblyopsidae* by specifying lognormal distributions such that 50% of the prior age of this divergence fell before the age of †*Massamorichthys wilsoni*, which is weakly supported as the oldest potential (though see Murray 1996, 2014; Murray et al. 2020) pan-aphredoderid in our Bayesian analysis of morphological data (supplementary fig. S4, Supplementary Material online). In both analyses, we set the rho prior to 1.0 to reflect that all percopsiforms are sampled in the dataset and the diversification rate prior to 0.05 with bounds of 1.0 and 0.0 following a previous estimate of the background acanthomorph diversification rate (Ghezelayagh et al. 2022). For the tip-dating analysis, we set the origin prior to the same specifications as those in the Bayesian analysis of morphological data. In the node-dating analysis, we set the origin prior to 97.2 Ma to reflect the minimum age of the oldest extinct forms (†*Xenyllion* spp., †*Boreohydrias dayi*, †*Cumbaaichthys oxyrhynchus*) assignable to the crown clade composed of *Percopsiformes* and *Polymixia* in our phylogeny built using morphological characters (supplementary fig. S4, Supplementary Material online), with bounds of 125.0 Ma (the base of the Barremian Stage of the Cretaceous, reflecting that no fossils assignable to crown *Acanthomorpha* are known from before the middle Barremian) and 66.02 Ma (the Cretaceous-Paleogene mass extinction, after which numerous representatives of *Percopsiformes*, etc., appear, see earlier). We ran both analyses each three times independently over 2.0 × 10^8^ generations with 5.0 × 10^7^ preburnin in BEAST v. 2.6.7 (Bouckaert et al. 2014; Bouckaert et al. 2019), used Tracer v. 1.7 to assess for convergence of the posteriors, and summarized the posterior tree sets using LogCombiner v. 2.6.6 and TreeAnnotator v. 2.6.6. A comparison of divergence time estimates found using the different calibration strategies is shown in supplementary fig. S7, Supplementary Material online.

### Ancestral State Reconstructions

**Fig. 1.**
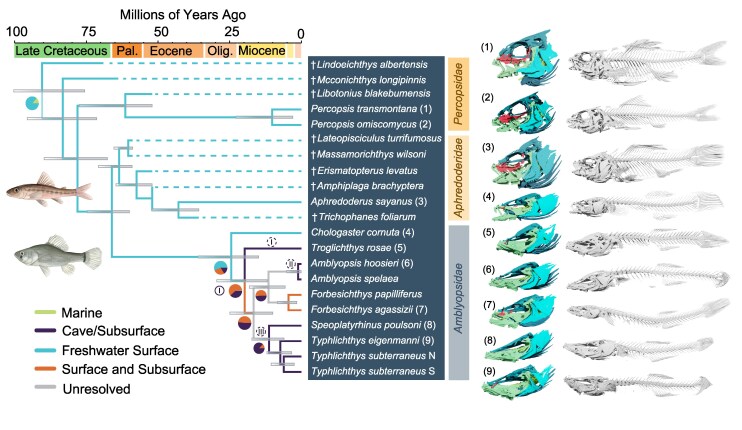
The timescale of amblyopsid cavefish evolution. Tip-dated Bayesian phylogeny of all recognized species of *Percopsiformes* including *Ambylopsidae* inferred from three sets of 50 randomly sampled ultraconserved elements using BEAST2. Colored branches indicate ancestral states inferred under a polymorphic character model in phytools, and pie charts indicate inferred states at nodes with uncertain (<80% probability of a single state) inferred ancestral states. To the right of the phylogeny are high-resolution computed tomography scan renders of percopsiform skulls and skeletons; numbers alongside tip labels indicate the corresponding scan. Colors of bone groups in scans indicate: suspensorium (green), circumorbital series (red), neurocranium (aquamarine), opercular series (light blue), branchiostegal series (dark blue), and pharyngeobranchial series (yellow). Roman numerals at nodes indicate potential cave colonization episodes based solely on the habitat ancestral state reconstruction (solid circle—minimum number of origins; dotted circles—maximum number of origins). Abbreviations: Pal., Paleocene; Olig., Oligocene; N, Northern; S, Southern. Daggers denote extinct species. Illustrations by Julia Johnson (https://www.lifesciencestudios.com/).

Based on our high-resolution computed tomography dataset, observations of preserved specimens, and the literature (Eigenmann 1909; Niemiller et al. 2013a; Armbruster et al. 2016; Hart et al. 2020), we compiled data on the habitats and anatomy of percopsiform fishes and conducted ancestral state reconstructions using the R package phytools (Revell 2012). We conducted two types of ancestral state reconstructions along the tip-dated Bayesian phylogeny inferred using the UCE sets. To accommodate the observation that some amblyopsids inhabit multiple regions that we treat as levels (e.g. the facultative cave dwellers in the genus *Forbesichthys*), we treated the habitat character as polymorphic. We fitted four candidate models (symmetrical and transient unordered, a general unordered model, and an equal transition rate model) of polymorphic character evolution and chose a favored model based on Akaike information criterion scores. Next, we conducted stochastic character mapping over 1,000 simulations and summarized the resulting posterior ancestral state reconstructions. We treated all other characters as monomorphic, used an equal rates model, and conducted stochastic character mapping over 1,000 simulations. The ancestral state reconstruction of habitat is shown in Fig. 1 and those for anatomical characters are shown in supplementary fig. S8, Supplementary Material online.

### Genomic Comparative Data Assembly

We downloaded the genome assemblies of 12 species of *Percopsiformes* from GenBank. We then used BUSCO v5.6.1 (Manni et al. 2021) on these genome assemblies to assess their completeness using the *Actinopterygii* odb10 database (3,640 genes, BUSCO_results), which also allowed us to assess the number of pseudogenes among BUSCO genes. For each species, we then downloaded all raw genomic reads available on the NCBI SRA database using the SRA-toolkit (https://github.com/ncbi/sra-tools). Whereas reads from only one specimen were retrieved for *Speoplatyrhinus poulsoni* and *Percopsis transmontana*, reads from multiple individuals were retrieved for the other species: two for *T. eigenmanni*, three for *Forbesichthys agassizii*, *Percopsis omiscomaycus*, *Amblyopsis hoosieri*, *A. spelaea*, and *Troglichthys rosae*, four for *Aphredoderus sayanus* and *Chologaster cornuta*, and five for *Forbesichthys papilliferus* and *Typhlichthys subterraneus* (supplementary figs. S9 and S10, Supplementary Material online). Reads were cleaned using fastp (Chen et al. 2018) and aligned to their corresponding genome assembly using BWA-mem (Vasimuddin et al. 2019). The average genome-wide coverage for each specimen, computed with SAMtools (Danecek et al. 2021), is in supplementary table S3, Supplementary Material online. We then used BCFtools (Danecek et al. 2021) to perform variant calling (“mpileup”), normalize indels (“norm”) and filter adjacent indels (“filter”). We used the resulting BCF file to (i) generate a consensus genome assembly for each specimen and (ii) generate consensus BUSCO gene sequences for each specimen. We then employed MUSCLE v. 5 (Edgar 2022) to align the protein sequences of BUSCO genes which were trimmed using TrimAl (Capella-Gutiérrez et al. 2009) and the option “automated1.” We concatenated trimmed alignments with AMAS (Borowiec 2016). Finally, we used IQ-TREE v. 2.2.0 (Minh et al. 2020b) to infer a maximum likelihood phylogeny using the general amino acid replacement matrix (Le and Gascuel 2008), empirical amino acid frequencies, and a discrete gamma model with four rate categories (LG + F + G4). We assessed support at nodes using 1,000 ultrafast bootstraps. These phylogenies are shown in supplementary fig. S9, Supplementary Material online.

### Mining for Vision-Related Genes

We defined a set of genes involved in light-perception in fishes as previously described by some of the authors of this study (Policarpo et al. 2021a). This dataset is composed of genes expressed in the eye, namely crystalline genes, genes involved in the transparency and refractive index of the eye (Delaye and Tardieu 1983), and genes involved in the phototransduction cascade, such as visual opsins. The dataset also comprises nonvisual opsins, which are expressed in various tissues (Davies et al. 2015). This dataset includes a total of 95 *Danio rerio* sequences representing 88 different genes, as some sequences are recent lineage specific duplicates (Policarpo et al. 2021a). We used *D. rerio* protein sequences of these genes as BLASTP (Camacho et al. 2009) queries against all *Percopsiformes* genome assemblies, with a threshold e-value of 1e−5. We extracted matching genomic sequences and extended them 100,000 bp upstream and downstream using SAMtools (Danecek et al. 2021). We then used EXONERATE (Slater and Birney 2005) to predict genes inside these regions. We manually inspected EXONERATE outputs to check for the presence of LoF mutations (loss of start codon, loss of stop codon, frameshift, premature stop codon, or splice site mutation) and their positions, as well as to extract the corresponding coding sequences (supplementary tables S3 to S5, Supplementary Material online).

We classified genes as (i) complete if a complete coding sequence was retrieved, (ii) incomplete if a truncated coding sequence was retrieved but with no LoF mutation, and (iii) pseudogenized if at least one LoF mutation was retrieved in the coding sequence. We assessed orthologous and paralogous relationships among genes using phylogenetic analyses. We translated gene coding sequences into protein sequences with EMBOSS transeq (Rice et al. 2000) after removing any frameshifts present and aligned sequences using MUSCLE v. 5 (Edgar 2022). We then used IQ-TREE2 to infer maximum likelihood trees for each gene after finding optimal models of nucleotide evolution using ModelFinder (Kalyaanamoorthy et al. 2017) and evaluated node support 1,000 ultrafast bootstraps (Hoang et al. 2018). Next, we used PAL2NAL (Suyama et al. 2006) to reverse translate protein alignments to codon alignments. We used these codon alignments and the maximum likelihood trees as input in the FitMG94 module implemented in HyPhy (Kosakovsky Pond et al. 2020) to compute dN/dS per branch. We discarded branches with too few synonymous mutations (dS < 0.01) or with a saturation of synonymous or nonsynonymous substitutions (dS or dN ≥ 1).

### LoF Substitutions Coverage and Genotyping

**Fig. 2.**
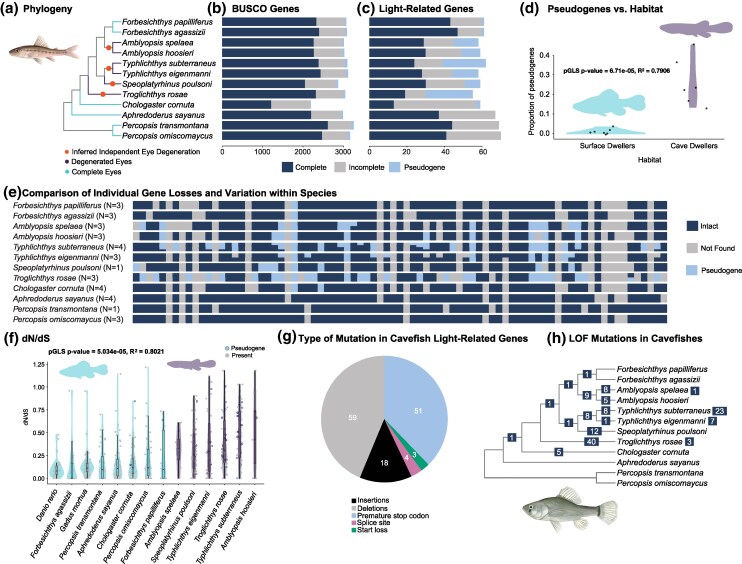
The comparative genomics of North American cavefishes. a) Phylogeny of *Percopsiformes* inferred from 874 ultraconserved elements and ASTRAL-III multispecies coalescent, showing inferred losses of vision based on comparative genomic analyses. The topology inferred using BUSCO genes is identical. Summary of all BUSCO b) and selected light-related c) genes recovered. Comparison d) of the proportions of light-related pseudogenes found in cave- and surface-dwelling percopsiform fishes, and two outgroups (*Danio rerio, Gadus morhua*). pGLS, phylogenetic generalized least-squares equation. Comparison e) of losses and pseudogenizations of light-related genes across and within species. Each row shows variation in gene presence and pseudogenization among sampled individuals of a given species. Comparison f) of dN/dS values for light-related genes in *Percopsiformes* and two outgroups (*D. rerio*, *G. morhua*). Pie chart g) shows a summary of mutation types found in light-related genes in amblyopsid cavefishes. Phylogeny in h) is the same as in a) and is annotated with an inferred history of LoF mutations. Numbers at nodes indicate LoF mutation counts at nodes, and numbers at tips indicate the total number of LoF mutations observed among individuals sampled for a species but not reconstructed as ancestral to the species itself. Illustrations by Julia Johnson (https://www.lifesciencestudios.com/).

We visualized BAM files resulting from the alignment of each specimen's raw reads to their corresponding genome assembly in the Integrative Genomics Viewer (IGV) (Robinson et al. 2011). For each vision gene, we recorded the number of reads supporting or not the observed LoF mutations identified in the genome assemblies. In addition, we verified the presence of additional LoF mutations which were not present in the genome assemblies. This led to the identification of seven additional LoF mutations in *T. subterraneus* specimens. For sites with at least one read supporting the presence and one read supporting the absence of LoF, we inferred the genotype from a cumulative binomial distribution (computed with the R function “pbinom”), which allowed us to calculate the probability that the allele supported by the minimum read count is a true allele (and assuming an equal probability of observing reads supporting both alleles). If the probability was less than 0.05, the genotype was assigned to homozygous for the allele supported by the maximum read count. Otherwise, the genotype was set to heterozygous (see Fig. 2, supplementary fig. S10, and S12, Supplementary Material online).

### Dating Relaxation of Purifying Selection on Vision Genes in Cave-Dwelling Species

We used a method described in a previous study (Policarpo et al. 2021a) to infer the number of generations since a gene had been pseudogenized. Briefly, we computed the probability that D genes among T genes contain at least one LoF mutation considering the length of each gene. This method requires an estimate of the LoF mutation rate per generation. Using a transition/transversion ratio in vision genes, 1.76, which we compute on vision genes with PAML v. 4.9 h (Yang et al. 2016), and codon usage in vision genes in the reference surface genome of *Percopsis transmontana*, we can compute the probability that a single nucleotide mutation in a coding sequence leads to a premature stop codon, hereafter called *p*_STOP_. Then, we can compute the probability, *p*_FS_, that a mutation is a frameshift as a function of *p*_STOP_:


$$p_{\mathrm{FS}}=[\mathrm{NFS}/\mathrm{NPSC}]\times p_{\mathrm{STOP}}$$

NFS is the number of observed frameshifts and NPSC is the number of observed premature stop codons. The probability of a single nucleotide mutation in a splice site (GT-AG) is


$$p_{\mathrm{SS}}=4\times [\mathrm{total}\;\mathrm{number}\;\mathrm{of}\;\mathrm{introns}/\mathrm{sum}\;\mathrm{of}\;\mathrm{CDS}\;\mathrm{lengths}]$$

The probability of the start codon loss is


$$p_{\mathrm{ML}}=3\times [\mathrm{number}\;\mathrm{of}\;\mathrm{vision}\;\mathrm{genes}/\mathrm{sum}\;\mathrm{of}\;\mathrm{CDS}\;\mathrm{lengths}]$$

and the probability of the stop codon loss, *p*_SL_, is


$$p_{\mathrm{SL}}=0.852\times p_{\mathrm{ML}}$$

where 0.852 is the proportion of mutations on a stop codon that leads to a nonstop codon.

The rate of stop codon loss is 23/27, where 27 is the number of different mutations in a codon and 4/27 is the proportion of mutations in a stop codon which leads to another stop codon.

Finally, we can estimate the loss-of-mutation rate, *µ*_LoF_, as a function of the single nucleotide mutation rate *µ* per site per generation:


$$\begin{array}{rl} \mu_{\mathrm{LoF}}= & [\;p_{\mathrm{FS}}+p_{\mathrm{STOP}}+p_{\mathrm{SS}}+p_{\mathrm{SL}}+p_{\mathrm{ML}}]\times \mu \\ = & [0.046+0.030+0.021+0.003+0.003]\times \mu \\ = & 0.103\times \mu \end{array}$$

The mutation rate *µ* was set to 0.5 × 10^−8^ or 1 × 10^−8^ according to recent estimates of the background mutation rate in teleost fishes (Bergeron et al. 2023). For this method, we assumed that all vision genes evolve as neutral sequences following cave colonization or that only vision genes found as a pseudogene in at least one cave species are evolving as neutral sequences. This assumption is based on several lines of evidence. First, nearly all candidate genes are consistently found to possess LoF mutations in cave-dwelling amblyopsids or other cave species (Fig. 2) (Policarpo et al. 2021a). Second, simulations of the distribution of LoF mutations conducted to assess the proportion of candidate genes evolving under relaxed selection favor the evolution of all or nearly all candidate genes under relaxed selection (Policarpo et al. 2021a). If there was selection for the loss of functional versions of these genes, we would expect LoF mutations to accumulate more rapidly than expected under neutral evolution. Whereas this might explain the loss of genes involved in the development of the eye (e.g. *pde6b*), the subsequent loss of vision genes once the eye has already been degenerated almost certainly resulted from neutral evolution. See also Fig. 4, supplementary fig. S11, Supplementary Material online.

**Fig. 3.**
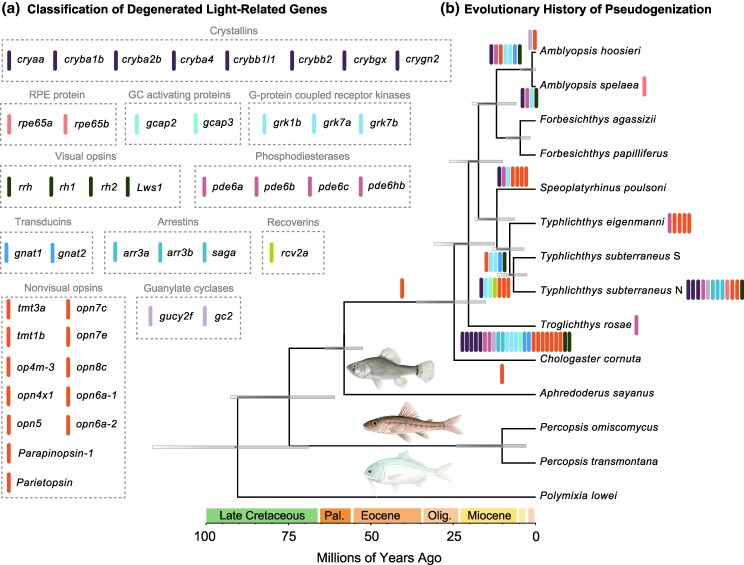
Evolutionary history of pseudogenization in *Amblyopsidae*. Node-dated Bayesian phylogeny generated using BEAST2 showing the distribution and inferred points of pseudogenization of light-related pseudogenes in *Percopsiformes* a, b). Bars along branches b) indicate inferred degeneration events among species, and bars to the right of tips indicate pseudogenes a) observed among individuals sampled for a species but not reconstructed as ancestral to the species itself. Illustrations by Julia Johnson (https://www.lifesciencestudios.com/).

**Fig. 4.**
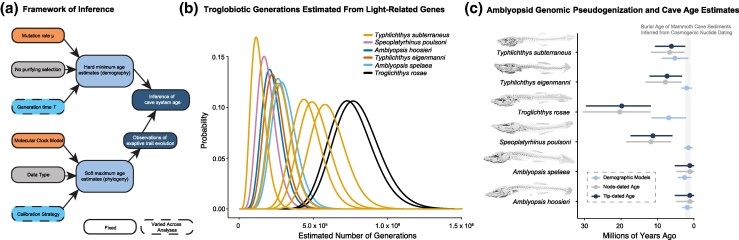
Cave age estimates using the pseudogene clock of cavefish genomes. Framework of inference incorporating genomic data, life history, and time-calibrated phylogenies a). Framework of inference for ages. b) Estimated number of generations for light-related gene pseudogenization in amblyopsid cavefishes c). Cave ages estimated using the cavefish pseudogenization generation time data and a three year long generation time for cavefishes, compared to estimates of node ages from tip-dated (Fig. 1) and node-dated (Fig. 2) phylogenies.

### Statistical Analyses

An R script file for reproducing analyses and figures of this study is available in GitHub (https://github.com/MaximePolicarpo/Amblyopsidae_Pseudogenes.git). We used the following R packages: patchwork v. 1.1.2, ape v. 5.7.1 (Paradis and Schliep 2019 ), dplyr v. 1.1.4 (Wickham et al. 2023a ), ggplot2 v. 3.4.0 (Wickham 2009), tidyverse v. 1.3.1 (Wickham et al. 2019 ), reshape2 v. 1.4.4 (Wickham 2007), adephylo v. 1.1.11 (Dray et al. 2023 ), phylobase v. 0.8.10 (Bolker et al. 2020 ), data.table v. 1.14.2 (Barrett et al. 2024), phytools v. 1.9.16 (Revell 2012 ), corrplot v. 0.92 (Wei et al. 2021), geiger v. 2.0.7 (Pennell et al. 2014), ggpubr v. 0.4.0 (Kassambara 2023), RColorBrewer v. 1.1.2 (Neuwirth 2022 ), scales v. 1.2.1 (Wickham et al. 2023b), phangorn v. 2.7.1 (Schliep 2011), and castor v. 1.7.6 (Louca 2024). We conducted phylogenetic generalized least-squares analysis using the R package caper v. 1.0.1 (Orme et al. 2013).

## Results

### Evolutionary Relationships of Amblyopsid Cavefishes

We inferred the evolutionary relationships of amblyopsid cavefishes and their relatives in *Percopsiformes* by integrating genomic data [874 ultraconserved elements (UCEs)] sequenced for all recognized living species and major lineages of the Southern Cavefish *Typhlichthys subterraneus* species complex (Niemiller et al. 2013b) with the percopsiform fossil record, including personal examination and comparison of fossils to high-resolution computed tomography scans of all living species (Figs. 1 to 3; supplementary figs. S1 to S9, Supplementary Material online; supplementary tables S1 and S2, Supplementary Material online). We also inferred a phylogeny using the concatenated set of BUSCO genes, which was identical to the species tree we inferred under a multispecies coalescent in ASTRAL-III (Zhang et al. 2018) (supplementary fig. S9, Supplementary Material online). Except for the placement of the early-diverging cavefish species *Troglichthys rosae*, the phylogeny of amblyopsids that we infer remains consistent across trees inferred using alternative partitioning methods (supplementary figs. S5, S6, and S9, Supplementary Material online). In our time-calibrated phylogeny of cavefishes, we estimate that crown *Amblyopsidae* originated in the late Oligocene 24.46 million years ago (95% highest posterior density interval: 14.91, 36.05 Ma), followed by the divergence of all major cavefish lineages between 19.8 and 11.46 million years ago (Fig. 1; supplementary figs. S4 and S7, Supplementary Material online). The long branch leading to crown amblyopsids from their common ancestry with the pirate perch *Aphredoderus sayanus* extends to 66.11 million years ago, just before the Cretaceous-Paleogene mass extinction. The diversification of amblyopsids breaks the pattern of species depauperacy that has persisted in percopsiform fishes over nearly 80 million years of Earth history (Fig. 1). All amblyopsid species last share common ancestry with their closest sister lineage over 1 million years ago (Fig. 1), demonstrating that these vertebrates represent truly ancient lineages rather than populations of surface-dwelling forms that have recently colonized caves as in populations of the model organism *Astyanax mexicanus* (Fumey et al. 2018; Herman et al. 2018).

Using our tip-dated phylogeny, we reconstructed the ancestral habitats of amblyopsid cavefishes and their closest relatives. We reconstruct an uncertain ancestral habitat for the backbone branches of amblyopsids (Fig. 1) exclusive of the Swampfish *Chologaster cornuta*, which occupies murky surface waters. These results conservatively indicate a minimum of one and maximum of three independent origins of obligate cave lifestyles among the genera *Troglichthys*, *Amblyopsis*, *Speoplatyrhinus*, and *Typhlichthys* (Fig. 1; supplementary fig. S8, Supplementary Material online). However, high-resolution computed tomography scans demonstrate that osteological features of the cavefish bauplan, including an elongated, flattened skull, reduced ossification of the circumorbital series, reduction of the supraoccipital crest, modification of the hyomandibular, elongation and shallowing of the thorax, and the reduction of pelvic fins, precede the evolution of obligate cavefishes (Fig. 1). These results are consistent with a previous study that used morphological characters to resolve amblyopsid phylogeny (Armbruster et al. 2016). *C. cornuta* and the facultative cave dwellers in the genus *Forbesichthys* possess less elongated skulls, more robustly built mandibles, and more ossified circumorbitals than obligate cave-dwelling amblyopsids (Fig. 1), even though *Forbesichthys* is nested within obligate cave-dwelling forms in our phylogeny (Fig. 1). However, *C. cornuta* and *Forbesichthys* possess considerably flatter skulls with more elongated rostra than the deep, robustly built crania of species in the genera *Aphredoderus* and *Percopsis*. The flattened skull and expanded cranial sensory pore system of *C. cornuta* have been proposed as adaptations to murky environments that were exapted in cave-dwelling amblyopsids for subterranean life (Poulson 1963). These observations underscore how a complex set of evolutionary transitions produced living amblyopsid cavefish diversity.

### The Genomic Basis of Degenerative Evolution in Ancient Cavefishes

Despite their usefulness for understanding the evolutionary history of *Amblyopsidae*, time-calibrated phylogenies might misrepresent the timescale of cave colonization in this clade. Theoretically, transitions from the surface to life in caves could have occurred anywhere along the branches leading to singleton cave species, rather than just at particular nodes in the tree. Consequently, the precise ages of these cave colonizations may not be captured by a time-calibrated phylogeny of all living species (Fig. 1).

To more thoroughly examine the timescale of cave adaptation, we investigated the genomic basis of degenerative evolution across 88 light-related genes in amblyopsids (Policarpo et al. 2021a,b) using 37 available genomes (supplementary table S3, Supplementary Material online). We searched for putative LoF mutations, including premature stop codons, losses of start and stop codons, frameshift mutations, and losses of intron splice sites in orthologs found across the sample of percopsiform genomes (Fig. 2; supplementary table S4, Supplementary Material online). Percopsiform fishes living in surface waters with functional eyes possess significantly fewer pseudogenes than their cave-dwelling relatives (Fig. 2a to d). Relative to the number of genes examined, no more than 3% of light-related genes are pseudogenized in surface-dwelling species (Fig. 2a to c). In contrast, in blind obligate cavefishes, pseudogenes are more numerous and showed higher variance across species (Fig. 2d). The lowest proportion of pseudogenes among cavefishes (10%) was found in a *Typhlichthys subterraneus* genome, and the highest (48%) was found in a *Troglichthys rosae* genome (supplementary tables S4 and S5, Supplementary Material online).

Our comparisons of pseudogenization in light-related genes of percopsiform fishes show that different lineages of cavefishes have degenerated different sets of genes involved in sight (Fig. 2e; supplementary fig. S10, Supplementary Material online; supplementary tables S4 and S5, Supplementary Material online). The degeneration of different gene sets and the presence of different LoF mutations in genes that have been degenerated in multiple cavefish lineages suggest that all four obligate cave-dwelling amblyopsid genera (*Amblyopsis, Speoplatyrhinus*, *Troglichthys*, and *Typhlichthys*) independently colonized subterranean ecosystems, even though two of these (*Speoplatyrhinus* and *Typhlichthys*) form a clade to the exclusion of all surface and facultative cave-dwelling amblyopsids. Our inference expands the number of inferred vision degeneration events in amblyopsids from a previous estimate based on ancestral state reconstruction of the presence of eyes across a phylogeny of *Percopsiformes* built using UCEs (Hart et al. 2020) and also highlights that different lineages of cavefishes have convergently lost the genomic basis of vision rather than experiencing pseudogenization at the same loci.

We find no evidence of any pseudogenization at the origin of the clade containing all amblyopsids except the surface-dwelling *Chologaster cornuta* or at any of the backbone nodes representing the common ancestors of ingroup obligate and facultative cave-dwelling species (Figs. 2e and 3), supporting our inference that the four obligate cave-dwelling amblyopsid lineages all independently colonized subterranean ecosystems (Fig. 1; supplementary figs. S1 to S3, Supplementary Material online). Higher average values and variance in dN/dS values for light-related genes in cavefishes relative to their surface-dwelling relatives are consistent with shifts in selective regimes for these genes as amblyopsids colonized caves, including relaxed selection, as well as bottleneck events associated with cave colonization (Fig. 2f).

Because the vast majority of mutations we detect in light-related genes in cavefishes are premature stop codons and deletions (Fig. 2g), our results support the hypothesis that modifications to the genetic architecture of sight in amblyopsids have primarily favored the degeneration of metabolically costly ocular structures. The highest numbers of pseudogenes (25) and LoF mutations (44) are found in the most deeply divergent (Fig. 1; supplementary figs. S4, S5, and S9, Supplementary Material online) cave-dwelling amblyopsid, the Ozark Cavefish *Troglichthys rosae* (supplementary tables S4 and S5, Supplementary Material online). Together with our tip- and node-dated phylogenies, this result suggests that the Ozark Cavefish is a long-lived troglomorphic lineage with no close surface relatives.

Among obligate cavefish lineages, we also find evidence of secondary degeneration of genes involved in vision. For example, *Amblyopsis spelaea* and *A. hoosieri* are reconstructed to share eight pseudogenes (seven of which are apomorphic to *Amblyopsis*), and individual specimens belonging to these species exhibit an additional two to five of these shared pseudogenes, respectively (Figs. 2e and h and 3b). We find evidence for only one LoF mutation occurring in the common ancestor of *Speoplatyrhinus poulsoni* and *Typhlichthys* (Fig. 2h; supplementary fig. S10, Supplementary Material online). This LoF mutation occurs in *opn7C*, a gene that we reconstruct as ancestrally pseudogenized in amblyopsids (Fig. 3). A total of 12 LoF mutations occur in the common ancestor of individuals of *S. poulsoni*, eight occur at the common ancestor of *Typhlichthys*, eight occur in ingroup branches of *T. eigenmanni*, and 31 LoF mutations occur in ingroup branches of *T. subterraneus.* In the common ancestor of the *T. subterraneus* species complex, we identified seven shared pseudogenes. This suggests that the ancestor of *T. subterraneus* was already a cavefish with degenerated eyes.

We also found that different lineages in the Southern Cavefish *Typhlichthys subterraneus* species complex have degenerated different sets of light-related genes (Fig. 2e and h). For example, 13 pseudogenes were found in the southern *T. subterraneus* lineage (exemplified by MLN0296; all already present in the common ancestor), but 25 (40% of the genes examined) were found in the northern lineage (Fig. 2e; supplementary fig. S10, Supplementary Material online; supplementary table S5, Supplementary Material online). These results are potentially consistent with the independent degeneration of the genetic bases of sight in cryptic species in the Southern Cavefish species complex (Niemiller et al. 2012, 2013b; Hart et al. 2024).

We infer that at least some modifications to the genome consistent with the degeneration of metabolically costly traits for life in cave environments preceded the evolution of obligate cave dwellers in a manner consistent with observations of amblyopsid soft tissue anatomy (Poulson 1963; Soares and Niemiller 2020) and osteology (Fig. 1; supplementary figs. S1 to S3, Supplementary Material online). We reconstruct the pseudogenization of one gene, *opn7c*, at the common ancestor of amblyopsids, the pseudogenization of *Parietopsin* and five LoF mutations at the common ancestor of *Chologaster cornuta* samples, and one loss-of-function mutation in the common ancestor of all amblyopsids excluding *C. cornuta* and *Troglichthys rosae* (Figs. 2h, and 3; supplementary fig. S10, Supplementary Material online). *Parietopsin* and *opn7c* both code for nonvisual opsins (Fig. 3), and the origins of LoF mutations in these genes prior to the divergence of cave-adapted amblyopsids cannot be directly used to infer “preadaptation” for life in caves. Nevertheless, these observations suggest that a few light-related genes may have been under relaxed selection before some amblyopsid lineages invaded caves. This result is consistent with Poulson's hypothesis that nocturnality in *C. cornuta,* the expansion of its neuromast system along a long, flattened skull for enhanced sensation in clogged freshwater habitats, and its reduced metabolic rate were ancestral features in amblyopsids that secondarily functioned as adaptations for life in caves (Poulson 1963). These observations suggest that the replicated evolution of cave-adapted species across 20 million years in *Amblyopsidae* via the independent and variable level of pseudogenization among a common set of vision-related genes in the dark (Fig. 3) is an example of exaptation (Gould and Vrba 1982) across physiological, skeletal, and soft-tissue traits that compose the ancestral amblyopsid phenotype.

### Amblyopsid Genomes Date the Age of Eastern North American Karst Caves

We used simulations to estimate the distribution and the maximum likelihood of the number of generations necessary to accumulate the observed number of pseudogenes among a set of light-related genes. Assuming a mutation rate of 1.0 × 10^−8^ per generation and that light-related genes are not under purifying selection and can accumulate LoF mutations in cavefishes, we estimate that different lineages of amblyopsids colonized caves between 114,000 and 768,000 generations ago (Fig. 4a and b). We estimate that the oldest cave colonization took place in *Troglichthys rosae* 7.5 × 10^5^ generations ago, followed by the colonization of caves by *Amblyopsis* 2.5 × 10^5^ generations ago, *Speoplatyrhinus poulsoni* 1.7 × 10^5^ generations ago, and *Typhlichthys subterraneus* between 1.14 and 5.78 × 10^5^ generations ago. Assuming a lower mutation rate of 5.0 × 10^−9^ instead of 1 × 10^−8^, these estimates become twice as large, and there is also wider variance in these generation estimates (supplementary table S8, Supplementary Material online). These results support the hypothesis that amblyopsid cavefishes independently colonized caves at different moments in deep time. We conservatively favor the estimates made using the higher mutation rate given that per-generation mutation rates in other paracanthopterygian fishes are between 1.0 and 2.0 × 10^−8^ per bp per generation (Matschiner et al. 2022).

## Discussion

Using our time-calibrated phylogenies and estimates of the number of generations in obligate cavefish lineages that show adaptations to subterranean life, we can infer a timescale of cave evolution in *Amblyopsidae*. Amblyopsid cavefish generation times are poorly known (Poulson 1963; Niemiller and Poulson 2010), but estimates range from 3 to 15 years (Poulson 1963; Niemiller and Poulson 2010; Niemiller et al. 2013b). This would suggest cave adaptations have occurred between 2.25 and 11.3 Ma in the most deeply divergent species *Troglichthys rosae* and between 342 ka to 1.71 Ma at minimum and 1.73 to 8.67 Ma at maximum for other cavefish lineages. Comparison of these estimates to our time-calibrated phylogenies shows that all of these estimates are younger than the most recent common ancestors of obligate cavefish lineages with other amblyopsids.

These results have three primary implications. First, because our estimates of the timing of cave adaptation in obligate subterranean amblyopsids are uniformly younger than our estimates of their divergence times from other amblyopsids, these results support the hypothesis that at least four amblyopsid lineages independently colonized caves after evolving from surface-dwelling ancestors. Second, they confirm the ancient ages of obligate cave-dwelling amblyopsids, contrasting with the recent (<500 ka, and perhaps <100 ka) settlement of caves observed in model cave populations of *Astyanax mexicanus* (Fumey et al. 2018; Herman et al. 2018; Policarpo et al. 2021b, 2024; Roback et al. 2025). At maximum, only a few light-related genes are pseudogenized in populations of *A. mexicanus*, and the reference genome of this species only includes one pseudogene (Policarpo et al. 2021b). In agreement with the hypothesized recent ages of cave colonization by *A. mexicanus* populations, most LoF mutations in light-related genes are not yet fixed and occur at low frequencies (Policarpo et al. 2021b). Third, these age estimates allow us to constrain the ages of ancient (older than 5 Ma) karstic ecosystems in eastern North America that are challenging to date using traditional geochronological techniques (Granger et al. 2001 ; Stock et al. 2005).

Our analyses demonstrate that phylogenies may be unreliable indicators of the age of cave colonization by amblyopsids. For example, our results indicate that a lag-time of at least eight million years may exist between the divergence of the oldest amblyopsid cavefish lineage *Troglichthys rosae* from other amblyopsids (Figs. 1 and 4; supplementary table S7, Supplementary Material online) and genomic evolution in *T. rosae* indicative of obligate cave life. Assuming that cave habitat availability was the primary constraint on amblyopsid cave colonization, we can identify a loose maximum bound for the age of a cave system based on the 95% highest posterior age densities in our phylogeny and a minimum bound based on the age of genomic signatures of cave adaptation. This “pseudogene clock” for landform origins is independent of assumptions (e.g. molecular clock and substitution model, prior ages given by fossils) made by molecular phylogenies (Fig. 3) and is dependent on the mutation rate *µ* and generation time *T*. Our calculations imply minimum ages of between 1.5 and 6.8 million years for eastern North American karst cave systems based on median estimates of generation times. Estimates vary between 2.51 and 11.51 Ma using 15-year generation times, 840 ka and 3.84 Ma based on 5-year generation times, and 500 ka and 2.3 Ma based on 3-year generation times (supplementary tables S6 and S7, Supplementary Material online). Assuming the exaptive nature of the amblyopsid bauplan for life in caves, soft upper bounds for the ages of cave systems based on our time-calibrated phylogenies are between 6 and 20 Ma. We used the relative number of examined genes that are pseudogenized in different cavefish lineages to estimate the ages of cave invasion by different clades. *T. rosae* has the most pseudogenized genes of those examined and was inferred to be oldest cave-adapted amblyopsid species, whereas species of *Amblyopsis* and *Speoplatyrhinus poulsoni*, which have the fewest pseudogenes, were inferred to be the most recent cave-adapted clades (Figs. 3 and 4c).

Geochronologic techniques for dating the origin of cave systems are largely limited to the past ∼5 Ma. For example, uranium-thorium dating of speleothems can provide dates up to ∼500 ka, carbon-dating of organic material can trace ages back to ∼50 ka, and estimates from burial ages of sediments using optically stimulated luminescence cosmogenic nuclide concentrations can be estimated up to ∼5 Ma (Stock et al. 2005; White 2007a,b). Cosmogenic nuclide dating has been used to constrain ages on sediments buried in caves in eastern North America, including in Mammoth Cave, Kentucky (3.25 ± 0.26 Ma) (Granger et al. 2001), and caves located in the Cumberland Plateau (5.68 ± 1.09 Ma) (Anthony and Granger 2004). Evidence of karst features during the late Miocene to early Pliocene is also supported by the fossil assemblage that characterizes the Gray Fossil Site, which is interpreted to represent an ancient sinkhole in the Tennessee Valley and Ridge physiographic province (7 to 3 Ma) (Clark et al. 2012). Karstification was likely older in the Florida carbonate platform, where carbonate rocks have experienced periodic subaerial exposure following the Eocene and a paleo-sinkhole preserves Oligocene-aged fossils (Patton 1969).

The available geochronologic constraints for the ages of cave systems in the Cumberland Plateau and Valley and Ridge geologic provinces are consistent with the minimum age bounds for the age of subterranean karst ecosystems inferred using the pseudogene clock (1.5 to 6.8 Ma) generated from observations of amblyopsid genomics and life history (Fig. 4). However, the genomics of amblyopsids, particularly *Troglichthys rosae*, indicate a history of karstic caves in eastern North America that stretches into the Miocene, > 10 Ma. Rather than representing very recent invasions of these habitats (Fumey et al. 2018), our analyses suggest that the exceptional and endangered (Eigenmann 1909; Christman et al. 2005; Niemiller et al. 2012, 2013b, 2021, 2023a,b; Gladstone et al. 2022; Benito et al. 2023; Hart et al. 2024) subterranean biodiversity of eastern North America accumulated over millions of years of Earth history.

Our results establish a macroevolutionary pathway toward cave adaptation in vertebrates that incorporates ancestral characteristics co-opted for life in subterranean habitats with extensive parallel degradation of the genetic bases of vision. This substantiates a hypothesis originally proposed at the beginning of the 20th century (Eigenmann 1909) that amblyopsids have convergently degenerated their vision. Amblyopsid cavefishes have convergently lost large portions of their light-related gene repertoire over the last 15 million years. Establishing the genomic context of these transitions allows us to discriminate between parallel colonization of cave systems that might otherwise be inferred as ancestral based on phylogeny or ancestral state reconstructions (Fig. 1; supplementary fig. S8, Supplementary Material online). Secondly, pinpointing the timescale of cave adaptation in blind amblyopsids provides new insight into the ages of the karst cave systems they invaded. Our results show the promise for the application of evolutionary histories to resolve ongoing questions in the earth sciences (Baker et al. 2014; Dawson et al. 2022) by drawing on observations from multiple facets of organismal biology to make robust inferences about the tempo and mode of interactions between the biosphere and geosphere.

## Supplementary Material

msaf185_Supplementary_Data

## Data Availability

Data associated with this manuscript are presented in the Supplementary Material online. CT scans are deposited on Morphosource under their corresponding YPM ICH numbers (the *Troglichthys rosae* is specimen TU Fish 22675 and found here: https://www.morphosource.org/concern/media/000031957?locale=en), and large data files, including sequence alignments and xml files needed to replicate the time-calibration analyses, will be deposited on Dryad: 10.5061/dryad.jwstqjqk9.
