# Supplementary material for: Convergent Evolution in Amblyopsid Cavefishes and the Age of Eastern North American Subterranean Ecosystems
Source: Mol Biol Evol. 2025 Aug 5;42(8):msaf185. doi: 10.1093/molbev/msaf185 (PMC12375919; doi:10.1093/molbev/msaf185)
Supplement: msaf185_Supplementary_Data [file msaf185_supplementary_data.pdf]

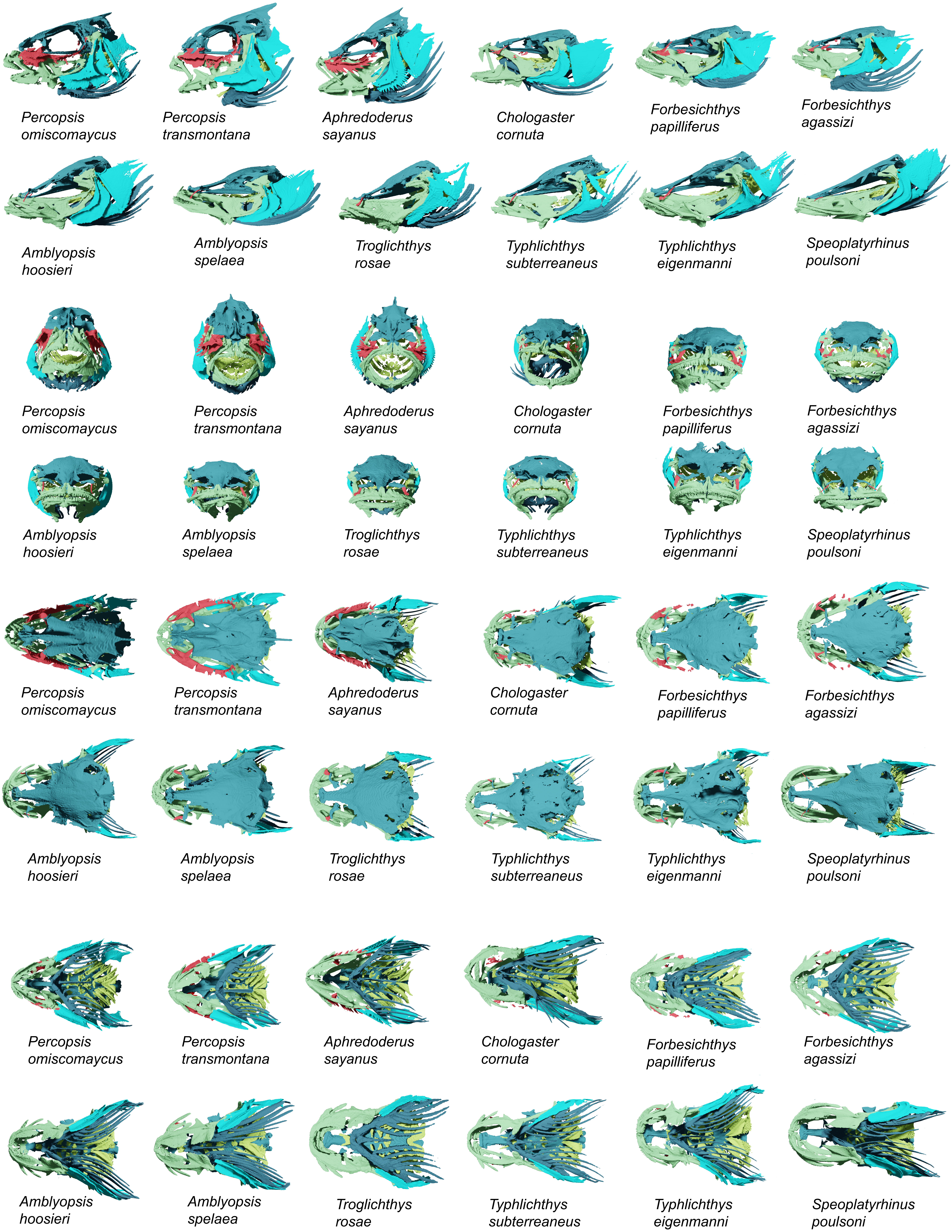

Percopsis omiscomaycus
Percopsis transmontana
Aphredoderus sayanus
Chologaster cornuta
Forbesichthys papilliferus
Forbesichthys agassizi
Amblyopsis hoosieri
Amblyopsis spelaea
Troglichthys rosae
Typhlichthys subterreaneus
Typhlichthys eigenmanni
Speoplatyrhinus poulsoni
Percopsis omiscomaycus
Percopsis transmontana
Aphredoderus sayanus
Chologaster cornuta
Forbesichthys papilliferus
Forbesichthys agassizi
Amblyopsis hoosieri
Amblyopsis spelaea
Troglichthys rosae
Typhlichthys subterreaneus
Typhlichthys eigenmanni
Speoplatyrhinus poulsoni
Percopsis omiscomaycus
Percopsis transmontana
Aphredoderus sayanus
Chologaster cornuta
Forbesichthys papilliferus
Forbesichthys agassizi
Amblyopsis hoosieri
Amblyopsis spelaea
Troglichthys rosae
Typhlichthys subterreaneus
Typhlichthys eigenmanni
Speoplatyrhinus poulsoni
Percopsis omiscomaycus
Percopsis transmontana
Aphredoderus sayanus
Chologaster cornuta
Forbesichthys papilliferus
Forbesichthys agassizi
Amblyopsis hoosieri
Amblyopsis spelaea
Troglichthys rosae
Typhlichthys subterreaneus
Typhlichthys eigenmanni
Speoplatyrhinus poulsoni

Figure S1. Percopsiform crania. Segmented and rendered microcomputed tomography scans of the crania of species of percopsiform fishes in left lateral (top two rows), anterior (next two rows), dorsal (next two rows), and ventral (bottom two rows) views. Colors of bone groups in scans indicate: suspensorium (green), circumorbital series (red), neurocranium (aquamarine), opercular series (light blue), branchiostegal series (dark blue), pharyngeobranchial series (yellow).

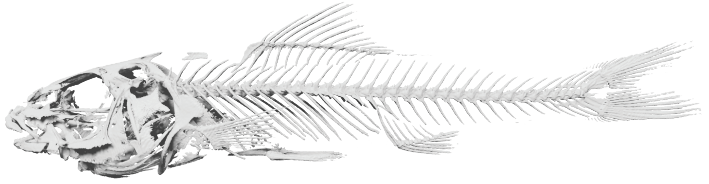

Percopsis omiscomycus

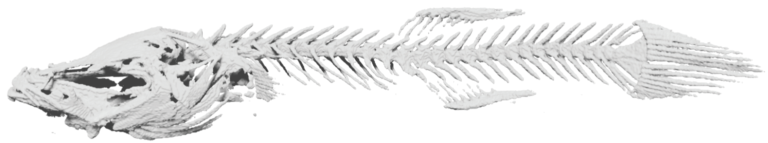

Troglichthys rosae

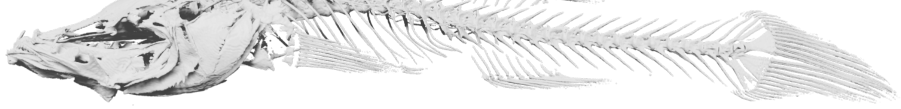

Amblyopsis spelaea

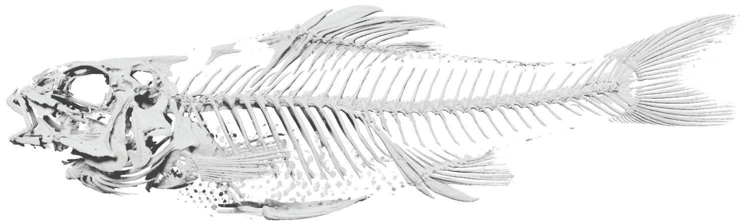

Percopsis transmontana

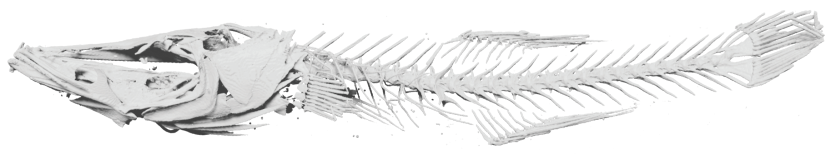

Speoplatyrhinus poulsoni

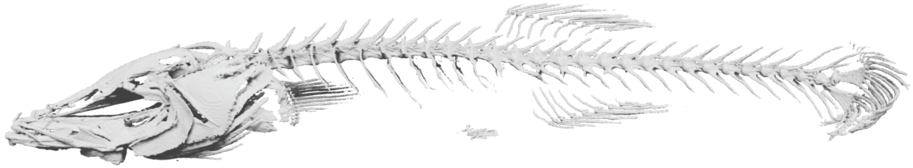

Amblyopsis hoosieri

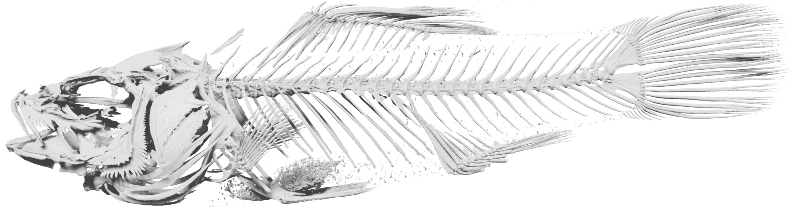

Aphredoderus sayanus

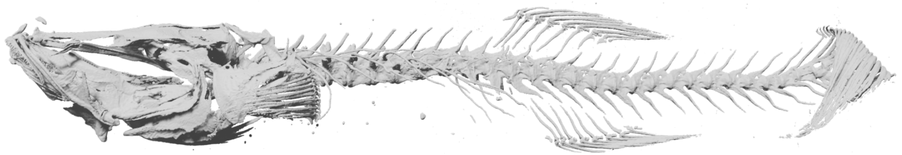

Typhlichthys eigenmannii

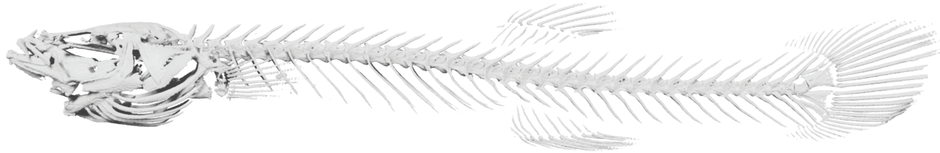

Forbesichthys agassizii

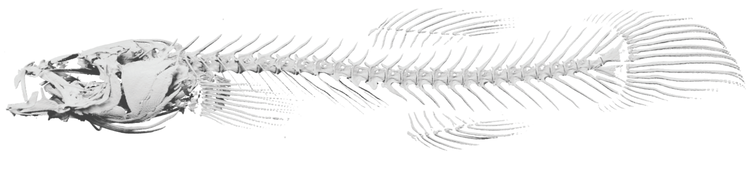

Chologaster cornuta

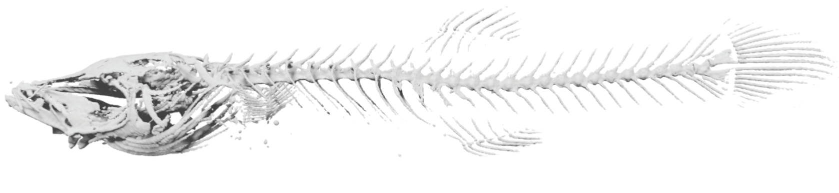

Typhlichthys subterraneus

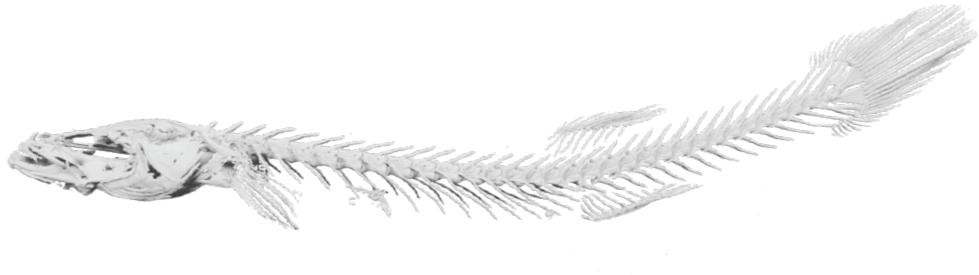

Forbesichthys papilliferus

Figure S2. Percopsiform skeletons. Segmented and rendered high-resolution computed tomography scans of the crania of species of percopsiform fishes in left lateral view.

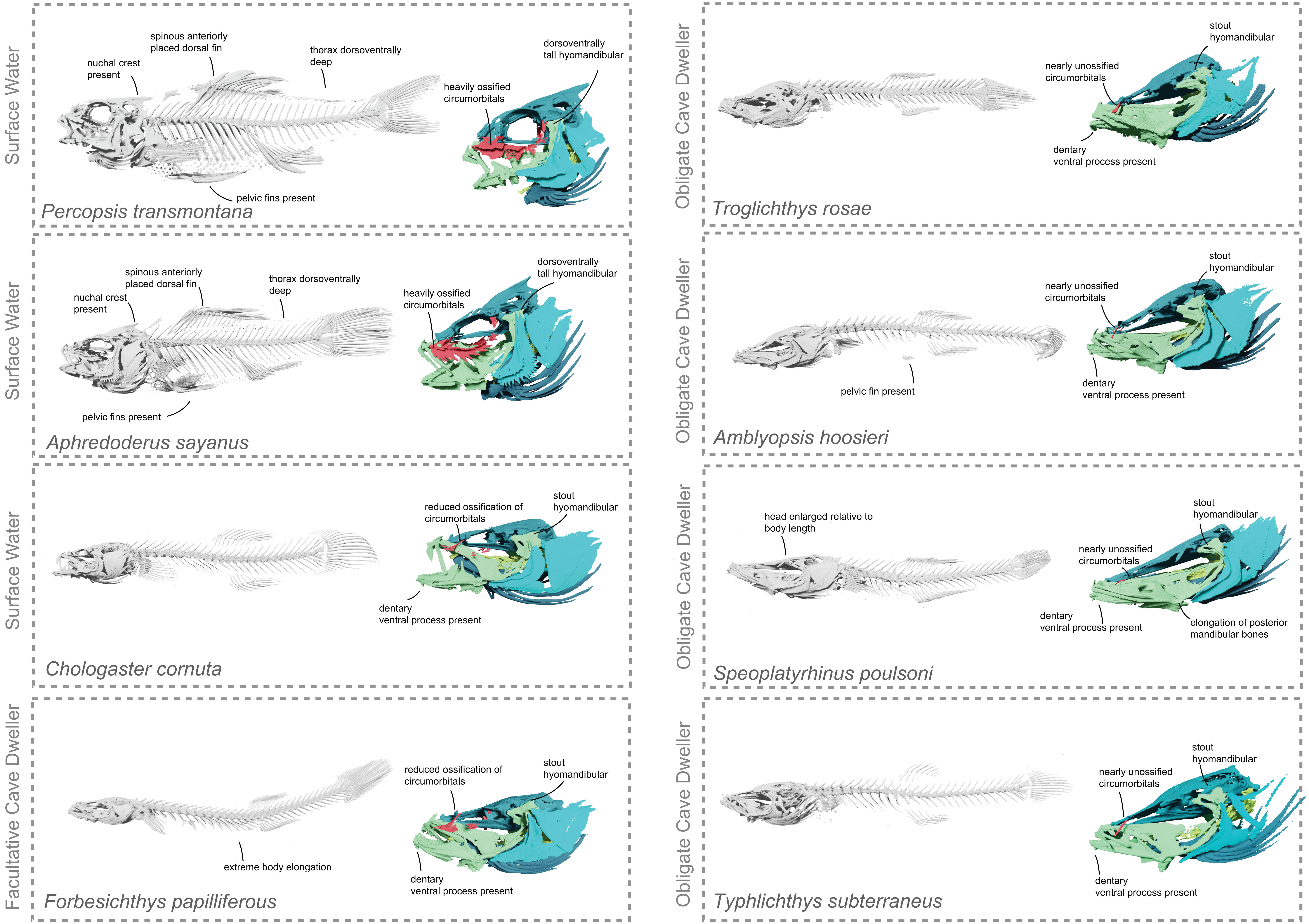
Surface Water
nuchal crest
present
spinous anteriorly
placed dorsal fin
thorax dorsoventrally
deep
dorsoventrally
tall hyomandibular
heavily ossified
circumorbitals
pelvic fins present
Percopsis transmontana
Surface Water
spinous anteriorly
placed dorsal fin
nuchal crest
present
thorax dorsoventrally
deep
dorsoventrally
tall hyomandibular
heavily ossified
circumorbitals
pelvic fins present
Aphredoderus sayanus
Surface Water
reduced ossification of
circumorbitals
stout
hyomandibular
dentary
ventral process present
Chologaster cornuta
Facultative Cave Dweller
reduced ossification of
circumorbitals
stout
hyomandibular
extreme body elongation
dentary
ventral process present
Forbesichthys papilliferous
Obligate Cave Dweller
stout
hyomandibular
nearly unossified
circumorbitals
dentary
ventral process present
Troglichthys rosae
Obligate Cave Dweller
stout
hyomandibular
nearly unossified
circumorbitals
pelvic fin present
dentary
ventral process present
Amblyopsis hoosieri
Obligate Cave Dweller
head enlarged relative to
body length
stout
hyomandibular
nearly unossified
circumorbitals
dentary
ventral process present
elongation of posterior
mandibular bones
Speoplatyrhinus poulsoni
Obligate Cave Dweller
stout
hyomandibular
nearly unossified
circumorbitals
dentary
ventral process present
Typhlichthys subterraneus

Figure S3. Comparative osteology of percopsiform crania and skeletons. Segmented and rendered high-resolution computed tomography scans of the crania and skeletons of selected species of percopsiform fishes in left lateral view, with key features indicated.

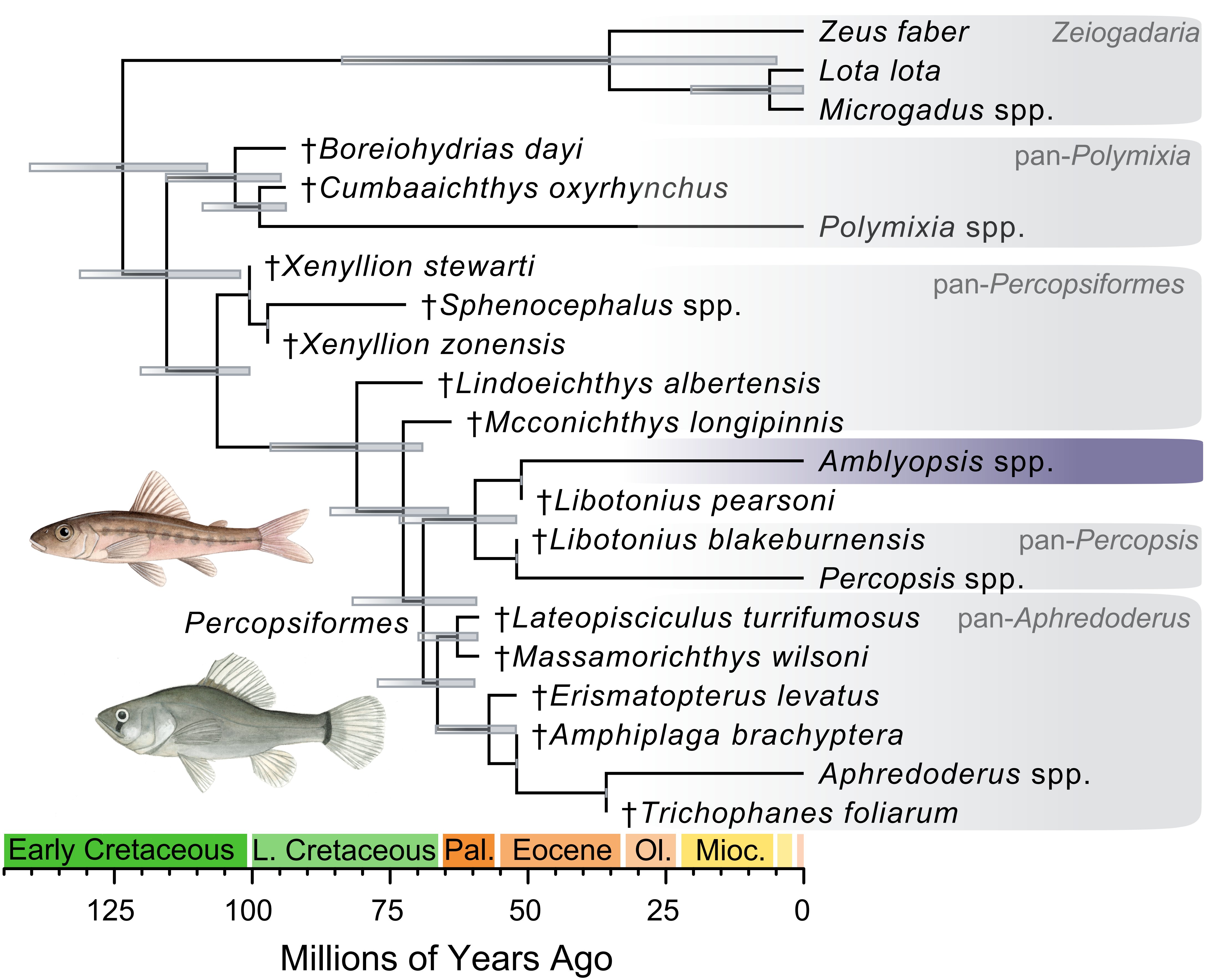
Zeus faber
Zeiogadaria
Lota lota
Microgadus spp.
†Boreiohydrias dayi
pan-Polymixia
†Cumbaaichthys oxyrhynchus
Polymixia spp.
†Xenyllion stewarti
pan-Percopsiformes
†Sphenocephalus spp.
†Xenyllion zonensis
†Lindoeichthys albertensis
†Mcconichthys longipinnis
Amblyopsis spp.
†Libotonius pearsoni
†Libotonius blakeburnensis
pan-Percopsis
Percopsis spp.
Percopsiformes
†Lateopisciculus turrifumosus
pan-Aphredoderus
†Massamorichthys wilsoni
†Erismatopterus levatus
†Amphiplaga brachyptera
Aphredoderus spp.
†Trichophanes foliarum
Early Cretaceous
L. Cretaceous
Pal.
Eocene
Ol.
Mioc.
125
100
75
50
25
0
Millions of Years Ago

Figure S4. Phylogenetic relationships of extinct percopsiform fishes. Tip-dated phylogeny of three extant and 12 extinct pan-percopsiforms with six outgroups generated from an analysis of morphological characters [2] under a Bayesian criterion in BEAST2. Bars at nodes indicate 95% highest posterior density intervals for node ages, and daggers (†) denote extinct species. Purple shaded color indicates the *Amblyopsidae*. Illustrations by Julia Johnson (https://www.lifesciencestudios.com/).

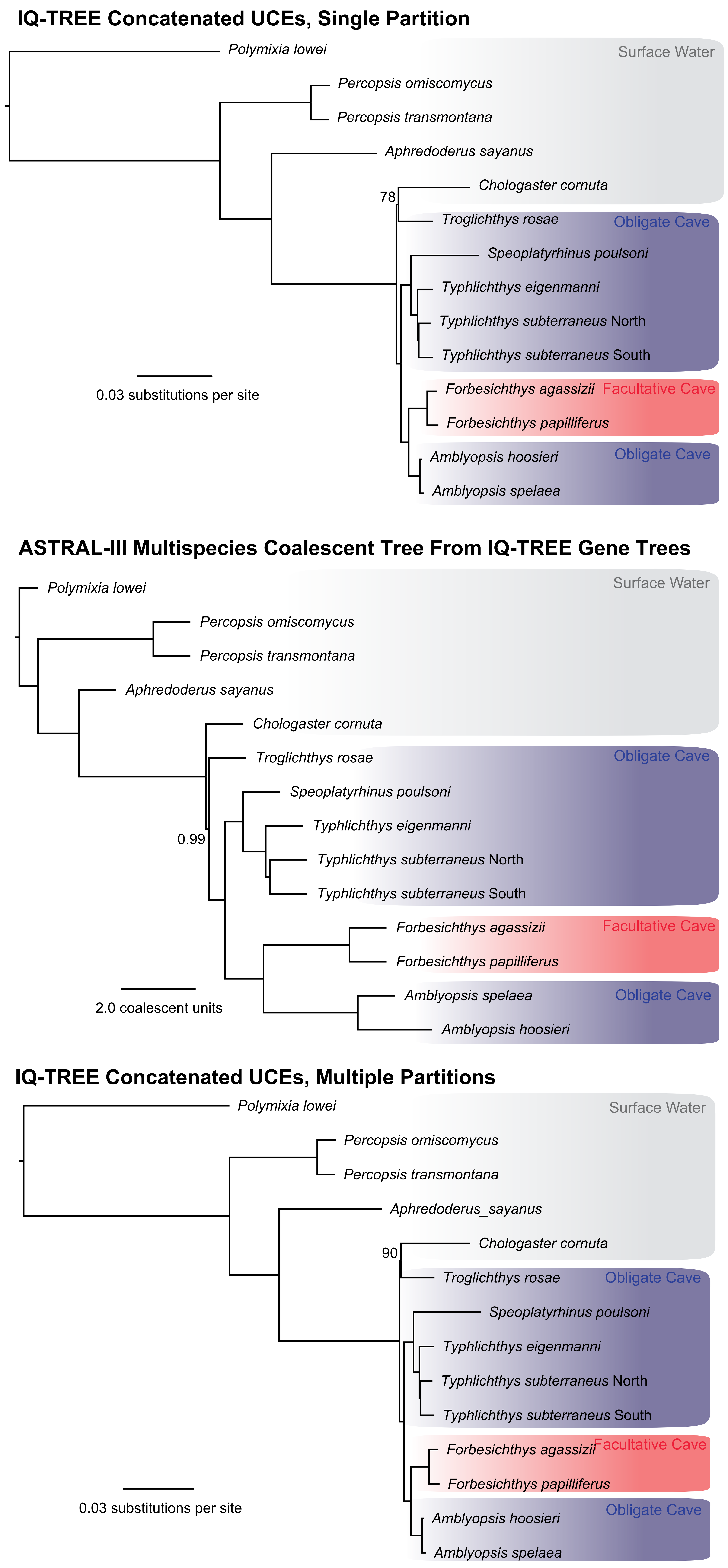

IQ-TREE Concatenated UCEs, Single Partition
Polymixia lowei
Surface Water
Percopsis omiscomycus
Percopsis transmontana
Aphredoderus sayanus
78
Chologaster cornuta
Troglichthys rosae
Obligate Cave
Speoplatyrhinus poulsoni
Typhlichthys eigenmanni
Typhlichthys subterraneus North
Typhlichthys subterraneus South
0.03 substitutions per site
Forbesichthys agassizii
Facultative Cave
Forbesichthys papilliferus
Amblyopsis hoosieri
Obligate Cave
Amblyopsis spelaea
ASTRAL-III Multispecies Coalescent Tree From IQ-TREE Gene Trees
Polymixia lowei
Surface Water
Percopsis omiscomycus
Percopsis transmontana
Aphredoderus sayanus
Chologaster cornuta
Troglichthys rosae
Obligate Cave
Speoplatyrhinus poulsoni
Typhlichthys eigenmanni
0.99
Typhlichthys subterraneus North
Typhlichthys subterraneus South
Forbesichthys agassizii
Facultative Cave
Forbesichthys papilliferus
Amblyopsis spelaea
Obligate Cave
2.0 coalescent units
Amblyopsis hoosieri
IQ-TREE Concatenated UCEs, Multiple Partitions
Polymixia lowei
Surface Water
Percopsis omiscomycus
Percopsis transmontana
Aphredoderus_sayanus
90
Chologaster cornuta
Troglichthys rosae
Obligate Cave
Speoplatyrhinus poulsoni
Typhlichthys eigenmanni
Typhlichthys subterraneus North
Typhlichthys subterraneus South
Forbesichthys agassizii
Facultative Cave
Forbesichthys papilliferus
0.03 substitutions per site
Amblyopsis hoosieri
Obligate Cave
Amblyopsis spelaea

Figure S5. Phylogenomics of *Percopsiformes*. Phylogenies of living percopsiform fishes generated using 874 ultraconserved element sequences and the maximum likelihood phylogenetics software IQ-TREE2 [32]. Top tree is from the analysis where all UCE sequences were concatenated and treated as a single partition, middle tree is the species tree created from 874 gene trees using the coalescent model in ASTRAL-III [35], and bottom tree is from the analysis where all UCE sequences were concatenated and partitioned according to a best-fit scheme found using PartitionFinder 2 [33]. Grey shaded regions indicate surface dwellers, red shaded regions indicate facultative cave dwellers, and purple shaded regions indicate obligate cave dwellers. Unless labeled, all nodes have bootstrap supports = 100 (top and bottom trees) or coalescent values = 1.0 (middle tree).

Figure S6. Concordance Factor Analysis. Plots showing associations among gene and site concordance factors and branch lengths calculated in IQ-TREE2. Except for the relationships of the *Typhlichthys* species complex and the root of *Amblyopsidae*, all nodes in the tree are well-supported across sites and loci.

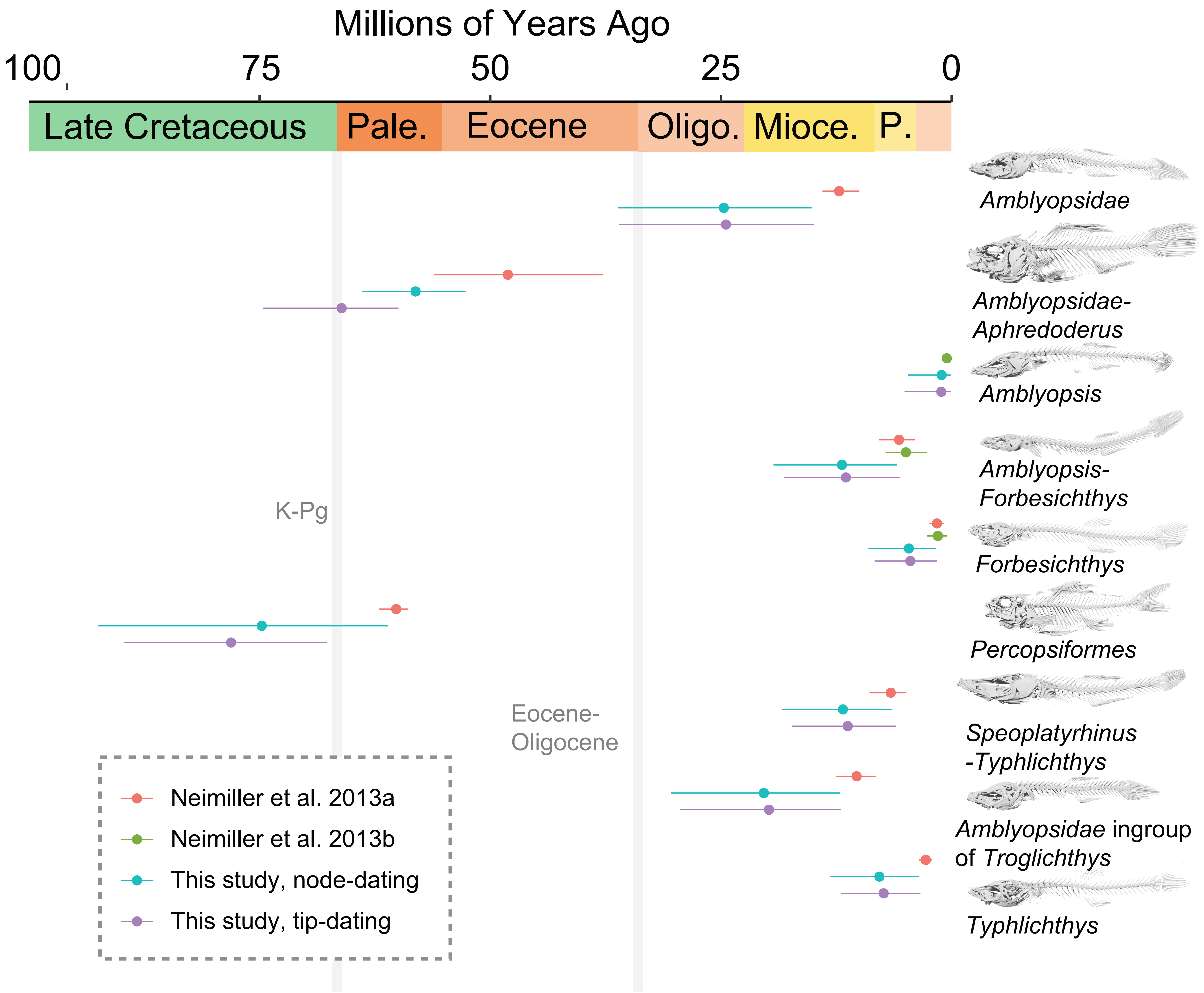

Millions of Years Ago
100
75
50
25
0
Late Cretaceous
Pale.
Eocene
Oligo.
Mioce.
P.
K-Pg
Eocene-
Oligocene
Amblyopsidae
Amblyopsidae-
Aphredoderus
Amblyopsis
Amblyopsis-
Forbesichthys
Forbesichthys
Percopsiformes
Speoplatyrhinus
-Typhlichthys
Amblyopsidae ingroup
of Troglichthys
Typhlichthys
Neimiller et al. 2013a
Neimiller et al. 2013b
This study, node-dating
This study, tip-dating

Figure S7. Divergence Time Estimate Comparisons. The divergence time estimates from our tip and node-dated Bayesian phylogenies of *Percopsiformes* compared to those found in previous studies leveraging legacy markers [39,78]. Bars indicate 95% highest posterior density intervals for node ages, and dots indicate median node ages.

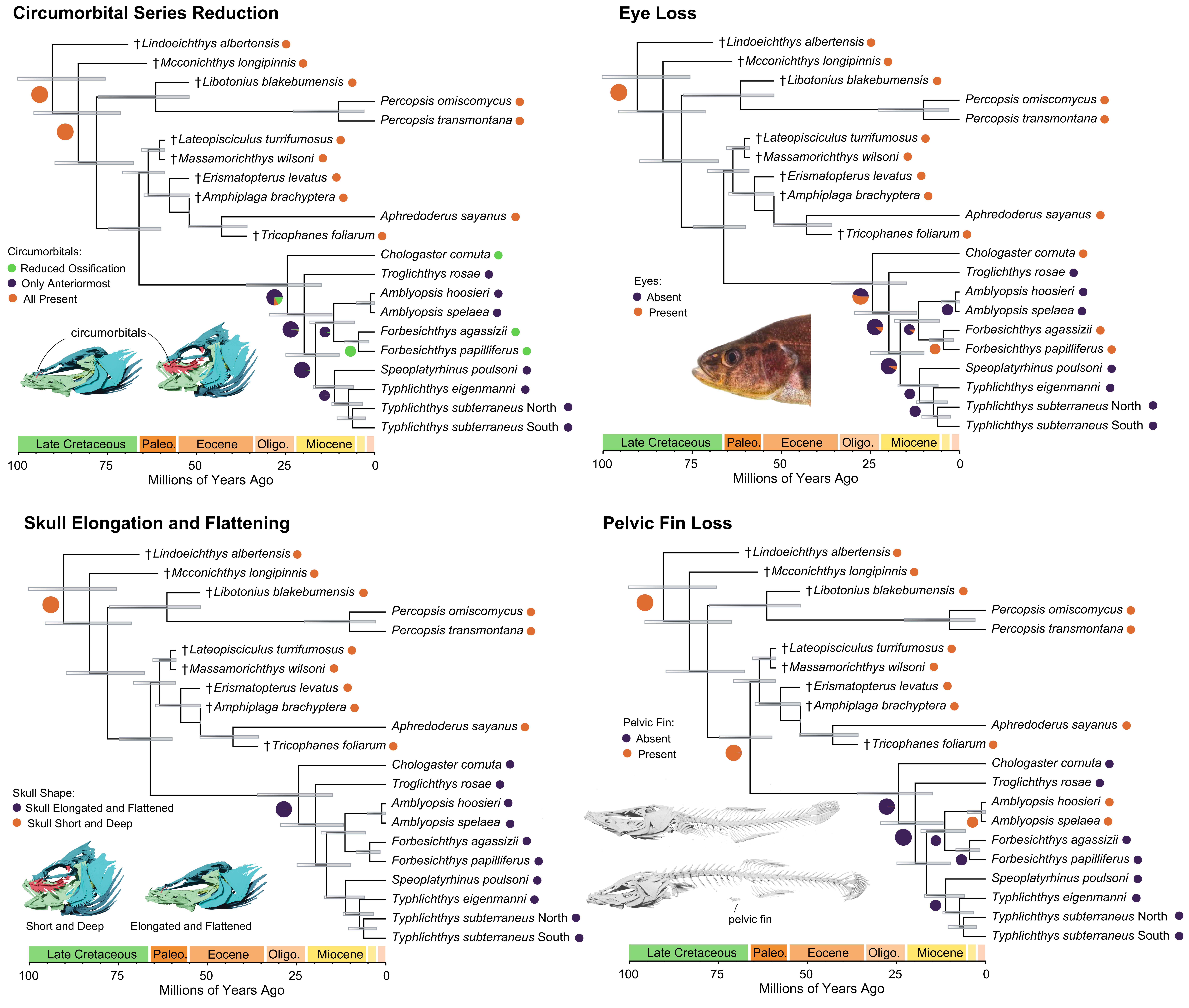

Circumorbital Series Reduction
†Lindoeichthys albertensis
†Mcconichthys longipinnis
†Libotonius blakebumensis
Percopsis omiscomycus
Percopsis transmontana
†Lateopisciculus turrifumosus
†Massamorichthys wilsoni
†Erismatopterus levatus
†Amphiplaga brachyptera
Aphredoderus sayanus
†Tricophanes foliarum
Chologaster cornuta
Troglichthys rosae
Amblyopsis hoosieri
Amblyopsis spelaea
Forbesichthys agassizii
Forbesichthys papilliferus
Speoplatyrhinus poulsoni
Typhlichthys eigenmanni
Typhlichthys subterraneus North
Typhlichthys subterraneus South
Circumorbitals:
Reduced Ossification
Only Anteriormost
All Present
circumorbitals
Late Cretaceous
Paleo.
Eocene
Oligo.
Miocene
100
75
50
25
0
Millions of Years Ago
Eye Loss
Eyes:
Absent
Present
Skull Elongation and Flattening
Skull Shape:
Skull Elongated and Flattened
Skull Short and Deep
Short and Deep
Elongated and Flattened
Pelvic Fin Loss
Pelvic Fin:
Absent
Present
pelvic fin

Figure S8. Ancestral State Reconstructions of Key Cavefish Osteological and Soft Tissue Features. SIMMAP ancestral state reconstructions conducted in phytools for circumorbital series reduction (top left), eye loss (top right), flat, elongated skulls (bottom left), and pelvic fin presence (bottom right). For all traits except eye loss (inferred as ancestral for all *Amblyopsidae* except *Chologaster cornuta*), the obligate cavefish condition (indicated in purple) is strongly inferred as ancestral to *Amblyopsidae*, with secondary reversals occurring within the clade. This contrasts with our hypothesis of cave colonization inferred using genomic data.

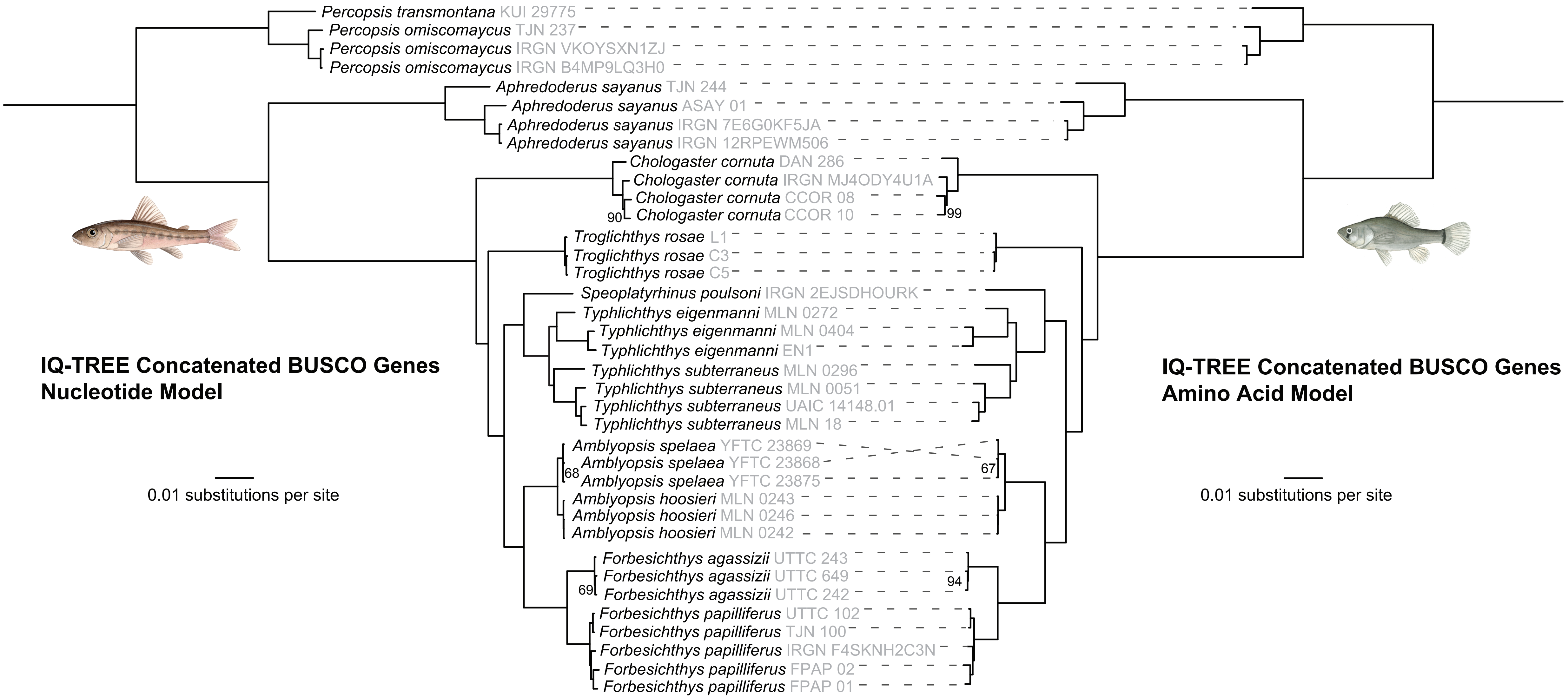
Percopsis transmontana KUI 29775
Percopsis omiscomaycus TJN 237
Percopsis omiscomaycus IRGN VKOYSXN1ZJ
Percopsis omiscomaycus IRGN B4MP9LQ3H0
Aphredoderus sayanus TJN 244
Aphredoderus sayanus ASAY 01
Aphredoderus sayanus IRGN 7E6G0KF5JA
Aphredoderus sayanus IRGN 12RPEWM506
Chologaster cornuta DAN 286
Chologaster cornuta IRGN MJ4ODY4U1A
Chologaster cornuta CCOR 08
90
Chologaster cornuta CCOR 10
99
Troglichthys rosae L1
Troglichthys rosae C3
Troglichthys rosae C5
Speoplatyrhinus poulsoni IRGN 2EJSDHOURK
Typhlichthys eigenmanni MLN 0272
Typhlichthys eigenmanni MLN 0404
Typhlichthys eigenmanni EN1
Typhlichthys subterraneus MLN 0296
Typhlichthys subterraneus MLN 0051
Typhlichthys subterraneus UAIC 14148.01
Typhlichthys subterraneus MLN 18
Amblyopsis spelaea YFTC 23869
Amblyopsis spelaea YFTC 23868
68
67
Amblyopsis spelaea YFTC 23875
Amblyopsis hoosieri MLN 0243
Amblyopsis hoosieri MLN 0246
Amblyopsis hoosieri MLN 0242
Forbesichthys agassizii UTTC 243
Forbesichthys agassizii UTTC 649
94
69
Forbesichthys agassizii UTTC 242
Forbesichthys papilliferus UTTC 102
Forbesichthys papilliferus TJN 100
Forbesichthys papilliferus IRGN F4SKNH2C3N
Forbesichthys papilliferus FPAP 02
Forbesichthys papilliferus FPAP 01
IQ-TREE Concatenated BUSCO Genes
Nucleotide Model
0.01 substitutions per site
IQ-TREE Concatenated BUSCO Genes
Amino Acid Model
0.01 substitutions per site

Figure S9. BUSCO Phylogenies. Phylogenies of *Percopsiformes* found using BUSCO genes. Unless noted, nodes are supported by bootstrap values equal to 100. Grey labels indicate sequence numbers. Illustration by Julia Johnson (https://www.lifesciencestudios.com/).

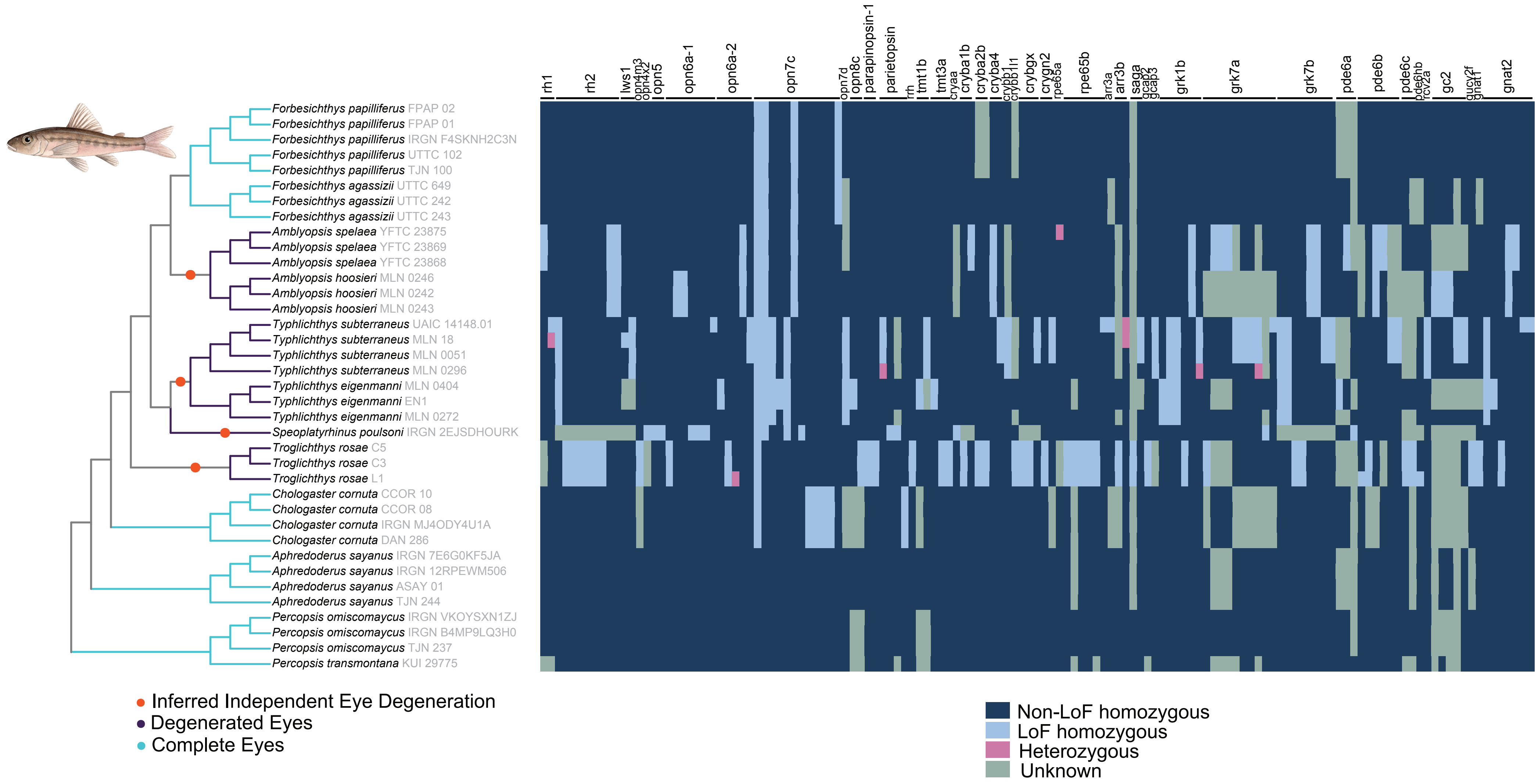
rh1
rh2
lws1
opn4m3
opn4x2
opn5
opn6a-1
opn6a-2
opn7c
opn7d
opn8c
parapinopsin-1
parietopsin
rrh
tmt1b
tmt3a
cryaa
cryba1b
cryba2b
cryba4
crybb1
crybb1l1
crybgx
crygn2
rpe65a
rpe65b
arr3a
arr3b
saga
gcap2
gcap3
grk1b
grk7a
grk7b
pde6a
pde6b
pde6c
pde6hb
rcv2a
gc2
gucy2f
gnat1
gnat2
Forbesichthys papilliferus FPAP 02
Forbesichthys papilliferus FPAP 01
Forbesichthys papilliferus IRGN F4SKNH2C3N
Forbesichthys papilliferus UTTC 102
Forbesichthys papilliferus TJN 100
Forbesichthys agassizii UTTC 649
Forbesichthys agassizii UTTC 242
Forbesichthys agassizii UTTC 243
Amblyopsis spelaea YFTC 23875
Amblyopsis spelaea YFTC 23869
Amblyopsis spelaea YFTC 23868
Amblyopsis hoosieri MLN 0246
Amblyopsis hoosieri MLN 0242
Amblyopsis hoosieri MLN 0243
Typhlichthys subterraneus UAIC 14148.01
Typhlichthys subterraneus MLN 18
Typhlichthys subterraneus MLN 0051
Typhlichthys subterraneus MLN 0296
Typhlichthys eigenmanni MLN 0404
Typhlichthys eigenmanni EN1
Typhlichthys eigenmanni MLN 0272
Speoplatyrhinus poulsoni IRGN 2EJSDHOURK
Troglichthys rosae C5
Troglichthys rosae C3
Troglichthys rosae L1
Chologaster cornuta CCOR 10
Chologaster cornuta CCOR 08
Chologaster cornuta IRGN MJ4ODY4U1A
Chologaster cornuta DAN 286
Aphredoderus sayanus IRGN 7E6G0KF5JA
Aphredoderus sayanus IRGN 12RPEWM506
Aphredoderus sayanus ASAY 01
Aphredoderus sayanus TJN 244
Percopsis omiscomaycus IRGN VKOYSXN1ZJ
Percopsis omiscomaycus IRGN B4MP9LQ3H0
Percopsis omiscomaycus TJN 237
Percopsis transmontana KUI 29775
Inferred Independent Eye Degeneration
Degenerated Eyes
Complete Eyes
Non-LoF homozygous
LoF homozygous
Heterozygous
Unknown

Figure S10. Loss of function mutations in individual percopsiform fishes. Figure shows the phylogeny of *Percopsiformes* reconstructed using BUSCO genes for 37 individuals (Genbank sequences labeled in grey) and types of loss-of-function mutations found for 43 vision-related genes that underwent pseudogenization in at least one cavefish species. Illustration by Julia Johnson (https://www.lifesciencestudios.com/).

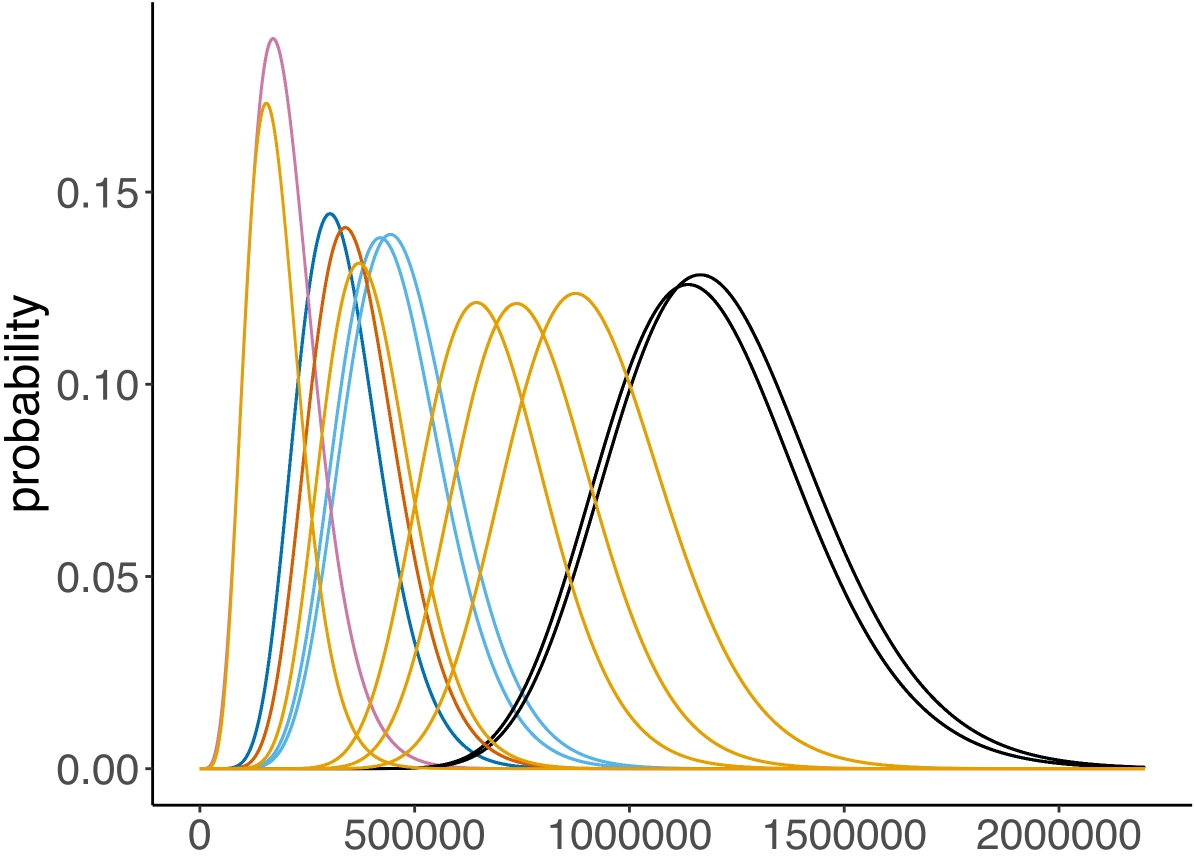
probability
0.15
0.10
0.05
0.00
0
500000
1000000
1500000
2000000

Figure S11. Generation Time Probabilities for cavefishes. Probability distribution curves for generation times estimated using only genes pseudogenized in at least one cavefish. Also see Table S6. X-axis indicates number of generations.

Mutations per gene per individual

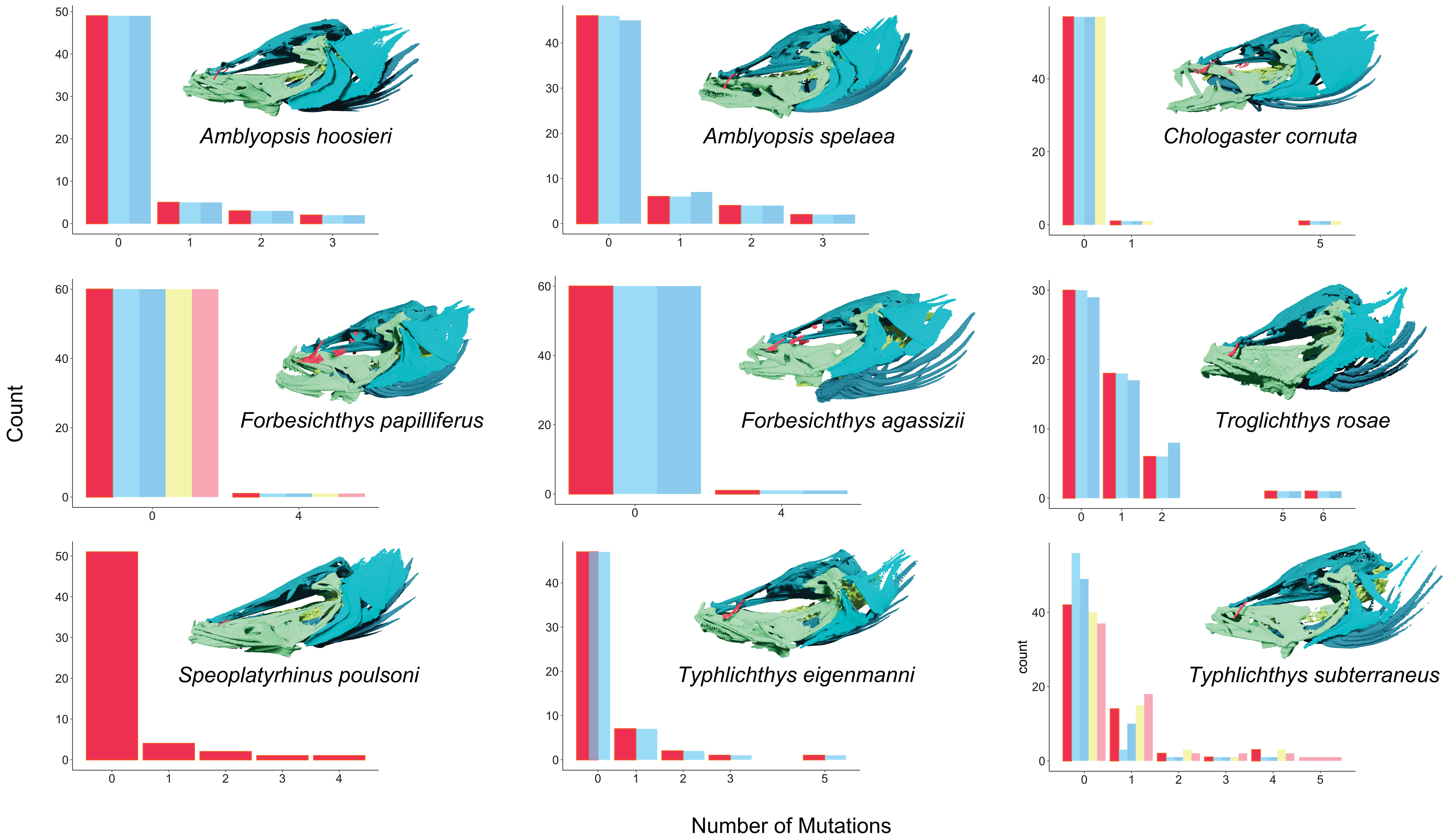

Figure S12. Variation in loss-of-function mutations across cavefish species. Graphs show variation in the number of loss-of-function mutations across individuals of the twelve known species of amblyopsids. Different colors for bars denote individuals for each species.

Table S1. CT Scan information. Specimen numbers, locality data, and lot counts for individual fishes examined using high-resolution computed tomography.

Taxon	Specimen	Lot Count	Locality	Tissue	Type
Aphredoderus sayanus	YPM ICH 31558	2	Rocky Mount. Tar River Drainage, Maple Creek @ Old Mill Road, Edgecombe County, NC	n/a	n/a
Percopsis transmontana	YPM ICH 9577	2	Columbia river, Tanner Creek Bypass, Oregon/Washington Border, Washington	n/a	n/a
Percopsis omiscomaycus	YPM ICH 17307	3	Muskinghum Drainage, Wakatoneta Creek at OH 79 crossing on Licking/Coshocton Co Line, Licking County, OH	UTTC 11163, UTTC 11164	n/a
Amblyopsis spelaea	YPM ICH 25294	7	Webster Cave, Breckinridge County, Kentucky, USA	YFTC 23868-75	hypotype
Amblyopsis hoosieri	YPM ICH 25305	1	Blue Springs Caverns, Lawrence County, IN	n/a	paratype
Amblyopsis hoosieri	YPM ICH 25304	2	Donaldson Cave, Spring Mill State Park, Lawrence County, IN	n/a	paratype

Typhlichthys subterraneus	YPM ICH 25594	2		Garner Spring Cove, Crow Creek/Tennesseee Drainage, Cave Stream, Franklin County, TN	n/a	n/a
Forbesichthys agassizii	YPM ICH 25310	7		spring fed ditch North of crossing with Morton Road, Todd County, Kentucky	n/a	n/a
Forbesichthys papilliferus	YPM ICH 25593	11		spring fed ditch North of crossing with Morton Road, Todd County, Kentucky	YFTC 023876-78	n/a
Chologaster cornuta	YPM ICH 25311	2		Colly Creek at NC 53, Bladen County, NC	YFTC 23849, YFTC 23850	n/a
Typhlichthys eigenmanni	UMMZ 150421	1		Bennett Spring Source, at Bennet State Park	n/a	n/a
Typhlichthys eigenmanni	UMMZ 156795	1		Camden River Cave near Hahatonka, Osage River Drainage, Missouri	n/a	n/a
Speoplatyrhinus poulsoni	UMMZ 197679	2		Lauderdale Key Cave, Alabama	n/a	n/a

Table S2. Ultraconserved element mining information. Genbank accession numbers for genomes from which we extracted ultraconserved elements for phylogenetic analysis.

Species	BioProject	BioSample
Amblyopsis hoosieri	PRJNA737769	SAMN20209475
Amblyopsis spelaea	PRJNA610650	SAMN14308568
Aphredoderus sayanus	PRJNA348720	SAMN05915031
Chologaster cornuta	PRJNA610650	SAMN14308572
Forbesichthys agassizii	PRJNA610650	SAMN14308580
Forbesichthys papilliferus	PRJNA610650	SAMN14308584
Percopsis omiscomycus	PRJNA348720	SAMN05915097
Percopsis transmontana	PRJEB12469	SAMEA4028770
Polymixia lowei	PRJNA348720	SAMN05915104
Speoplatyrhinus poulsoni	PRJNA610650	SAMN14308586
Troglichthys rosae	PRJNA610650	SAMN14308599
Typhlichthys eigenmanni	PRJNA610650	SAMN14308592
Typhlichthys subterraneus	PRJNA610650	SAMN14308618
Typhlichthys subterraneus	PRJEB12469	SAMEA4028771

Table S3. Mean Genome Coverage Per Specimen.

Species	Sample ID	GenBank BioSample	Mean read depth
Amblyopsis hoosieri	MLN 0242	SAMN20209475	32.914
Amblyopsis hoosieri	MLN 0243	SAMN47174500	31.6036
Amblyopsis hoosieri	MLN 0246	SAMN20209477	37.391
Amblyopsis rosae	C3	SAMN20209505	37.4894
Amblyopsis rosae	C5	SAMN20209506	34.2265
Amblyopsis rosae	L1	SAMN20209507	34.2636
Amblyopsis spelaea	YFTC 23868	SAMN20209478	30.8339
Amblyopsis spelaea	YFTC 23869	SAMN20209479	27.3974
Amblyopsis spelaea	YFTC 23875	SAMN20209480	31.5756
Aphredoderus sayanus	ASAY 01	SAMN20209483	33.8516
Aphredoderus sayanus	IRGN 12RPEWM506	SAMN20209482	30.6338
Aphredoderus sayanus	IRGN 7E6G0KF5JA	SAMN20209481	39.2077
Aphredoderus sayanus	TJN 244	SAMN20209484	23.5689
Chologaster cornuta	CCOR 08	SAMN20209485	34.2061
Chologaster cornuta	CCOR 10	SAMN20209486	36.9205
Chologaster cornuta	DAN 286	SAMN20209487	20.1177
Chologaster cornuta	IRGN MJ4ODY4U1A	SAMN19789269	37.4254
Forbesichthys agassizii	UTTC 242	SAMN20209490	35.2239
Forbesichthys agassizii	UTTC 243	SAMN20209491	41.739
Forbesichthys agassizii	UTTC 649	SAMN20209492	35.9584
Forbesichthys papilliferus	FPAP 01	SAMN20209494	27.231
Forbesichthys papilliferus	FPAP 02	SAMN20209495	21.5146
Forbesichthys papilliferus	IRGN F4SKNH2C3N	SAMN20209496	34.1706
Forbesichthys papilliferus	TJN 100	SAMN20209497	29.7276
Forbesichthys papilliferus	UTTC 102	SAMN20209493	31.524
Percopsis omiscomaycus	IRGN B4MP9LQ3H0	SAMN20209498	36.3774
Percopsis omiscomaycus	IRGN VKOYSXN1ZJ	SAMN20209499	22.4277
Percopsis omiscomaycus	TJN 237	SAMN20209500	32.8414
Percopsis transmontana	KU KUI 29775	SAMEA4028770	34.417
Typhlichthys eigenmanni	EN1	SAMN47174533	38.2493
Typhlichthys eigenmanni	MLN 0404	SAMN20209509	32.989
Typhlichthys subterraneus	MLN 0051	SAMN20209512	28.354
Typhlichthys subterraneus	MLN 0272	SAMN20209513	51.7697
Typhlichthys subterraneus	MLN 0296	SAMN20209514	34.3673
Typhlichthys subterraneus	MLN 18	SAMN20209515	32.7368
Typhlichthys subterraneus	UAIC 14148 01	SAMEA4028771	29.6567
Speoplatyrhinus poulsoni	IRGN 2EJSDHOURK	SAMN20209504	28.0822

Table S4. Pseudogene summary statistics. Pseudogene and loss-of-function mutation counts among the candidate gene set for percopsiform species and outgroups.

Species	Complete	Incomplete	Pseudogene	Total	Pseudogene Proportion	Bp	Number of LoF mutations	LoF mutations per sequence length
Aphredoderus sayanus	37	30	0	67	0	58314	0	0.000000e+00
Danio rerio	95	0	0	95	0	103809	0	0.000000e+00
Percopsis omiscomaycus	41	29	0	70	0	62676	0	0.000000e+00
Percopsis transmontana	44	25	0	69	0	61308	0	0.000000e+00
Forbesichthys agassizii	47	13	1	61	0.01639344	58287	4	6.862594e-05
Forbesichthys papilliferus	43	17	1	61	0.01639344	59367	4	6.737750e-05
Chologaster cornuta	13	44	2	59	0.03389831	40901	6	1.466957e-04
Speoplatyrhinus poulsoni	30	21	8	59	0.13559322	49492	15	3.030793e-04
Amblyopsis hoosieri	30	19	10	59	0.16949153	51839	17	3.279384e-04
Amblyopsis spelaea	29	16	13	58	0.22413793	49737	21	4.222209e-04
Typhlichthys eigenmanni	28	16	14	58	0.24137931	53052	23	4.335369e-04
Typhlichthys subterraneus	24	15	23	62	0.37096774	53463	37	6.92E-04
Troglichthys rosae	19	11	26	56	0.46428571	46391	44	9.484598e-04

Table S5. Pseudogene summary statistics II. Pseudogene and loss-of-function mutation counts among the candidate gene set for multiple individuals of each percopsiform species.

Species and Specimen	Intact	Pseudogene	Total	Pseudogene Proportion	Bp	Number of LoF mutations	LoF mutations per sequence length
Aphredoderus sayanus ASAY 01	67	0	67	0	58314	0	0
Aphredoderus sayanus lRGN 12RPEWM506	67	0	67	0	58314	0	0
Typhlichthys subterraneus MLN 0272	56	6	62	0.09677419	53463	12	2.24E-04
Aphredoderus sayanus lRGN 7E6GOKF5JA	67	0	67	0	58314	0	0
Speoplatyrhinus poulsoni lRGN 2EJSDHOURK	51	8	59	0.13559322	49492	15	3.03E-04
Aphredoderus sayanus TJN 244	67	0	67	0	58314	0	0
Amblyopsis hoosieri MLN 0242	49	10	59	0.16949153	51839	17	3.28E-04
Percopsis omiscomaycus lRGN B4MP9LQ3HO	70	0	70	0	62676	0	0
Amblyopsis hoosieri MLN 0243	49	10	59	0.16949153	51839	17	3.28E-04
Percopsis omiscomaycus lRGN VKOYSXN1ZJ	70	0	70	0	62676	0	0
Amblyopsis hoosieri MLN 0246	49	10	59	0.16949153	51839	17	3.28E-04

Percopsis omiscomaycus TJN 237	70	0	70	0	62676	0	0
Typhlichthys eigenmanni EN1	47	11	58	0.18965517	53052	19	3.58E-04
Percopsis transmontana KU KUl 29775	69	0	69	0	61308	0	0
Typhlichthys eigenmanni MLN 0404	47	11	58	0.18965517	53052	19	3.58E-04
Forbesichthys agassizii UTTC 242	60	61		0 01639344	58287	4	6.86E-05
Amblyopsis spelaea YFTC 23868	46	12	58	0.20689655	49737	20	4.02E-04
Forbesichthys agassizii UTTC 243	60	1	61	0.01639344	58287	4	6.86E-05
Amblyopsis spelaea YFTC 23869	46	12	58	0.20689655	49737	20	4.02E-04
Forbesichthys agassizii UTTC 649	60	1	61	0.01639344	58287	4	6.86E-05
Typhlichthys subterraneus MLN 0296	49	13	62	0.20967742	53463	19	3.55E-04
Forbesichthys papilliferus FPAP 01	60	1	61	0.01639344	59367	4	6.74E-05
Amblyopsis spelaea YFTC 23875	45	13	58	0.22413793	49737	21	4.22E-04
Forbesichthys papilliferus FPAP 02	60	1	61	0.01639344	59367	4	6.74E-05
Typhlichthys subterraneus MLN 0051	42	20	62	0.32258065	53463	33	6.17E-04
Forbesichthys papilliferus lRGN F4SKNH2C3N	60	1	61	0.01639344	59367	4	6.74E-05

Typhlichthys subterraneus MLN 18	40	22	62	0.35483871	53463	36	6.73E-04
Forbesichthys papilliferus TJN 100	60	1	61	0.01639344	59367	4	6.74E-05
Forbesichthys papilliferus UTTC 102	60	1	61	0.01639344	59367	4	6.74E-05
Typhlichthys subterraneus UAIC 14148 01	37	25	62	0.40322581	53463	41	7.67E-04
Chologaster cornuta CCOR 08	57	2	59	0.03389831	40901	6	1.47E-04
Troglichthys rosae C3	30	26	56	0.46428571	46391	41	8.84E-04
Chologaster cornuta CCOR 10	57	2	59	0.03389831	40901	6	1.47E-04
Troglichthys rosae C5	30	26	56	0.46428571	46391	41	8.84E-04
Chologaster cornuta DAN 286	57	2	59	0.03389831	40901	6	1.47E-04
Troglichthys rosae L1	29	27	56	0.48214286	46391	44	9.48E-04
Chologaster cornuta lRGN MJ40DY4U1A	57	2	59	0.03389831	40901	6	1.47E-04

Table S6. Generation Times I. Generation times estimated using all candidate genes. 'Best GenNb" : Maximum likelihood date. "MinGen" and "MaxGen) correspond to the lower bound and higher bound (p-value of 0.05) of the datation.

	Species and Specimen	MaxProba	MinGen p<0.05	MaxGen p<0.05
114071	*Typhlichthys subterraneus* MLN 0272	0.1689847	55931	203001
167871	*Speoplatyrhinus poulsoni* lRGN 2EJSDHOURK	0.1501026	94491	272021
204271	*Amblyopsis hoosieri* MLN 0242	0.1372445	125611	310531
204271	*Amblyopsis hoosieri* MLN 0243	0.1372445	125611	310531
204271	*Amblyopsis hoosieri* MLN 0246	0.1372445	125611	310531
222191	*Typhlichthys eigenmanni* EN1	0.1325695	141191	329591
222191	*Typhlichthys eigenmanni* MLN 0404	0.1325695	141191	329591
261231	*Amblyopsis spelaea* YFTC 23868	0.1283738	170751	379281
261231	*Amblyopsis spelaea* YFTC 23869	0.1283738	170751	379281
263721	*Typhlichthys subterraneus* MLN 0296	0.1236226	177021	375101
286001	*Amblyopsis spelaea* YFTC 23875	0.124762	191541	407541
436491	*Typhlichthys subterraneus* MLN 0051	0.1078646	325861	570041
491171	*Typhlichthys subterraneus* MLN 18	0.1054141	373411	631831
578541	*Typhlichthys subterraneus* UAIC 14148.01	0.1028473	449371	730961
728131	*Troglichthys rosae* C3	0.1064155	564391	921941
728131	*Troglichthys rosae* C5	0.1064155	564391	921941
767681	*Troglichthys rosae* L1	0.1062139	597961	968071

Table S7. Generation Times II. Generation times estimated using only genes pseudogenized in at least one cavefish. 'Best GenNb" : Maximum likelihood date. "MinGen" and "MaxGen) correspond to the lower bound and higher bound (p-value of 0.05) of the datation.

Best GenNb	Species and Specimen	MaxProba	MinGen p<0.05	MaxGen p<0.05
154651	*Typhlichthys subterraneus* MLN 0272	0.1731032	75211	276731
170361	*Speoplatyrhinus poulsoni* lRGN 2EJSDHOURK	0.1899124	74131	327141
302941	*Amblyopsis hoosieri* MLN 0242	0.1443643	183731	465481
302941	*Amblyopsis hoosieri* MLN 0243	0.1443643	183731	465481
302941	*Amblyopsis hoosieri* MLN 0246	0.1443643	183731	465481
338531	*Typhlichthys eigenmanni* EN1	0.140771	211741	508521
338531	*Typhlichthys eigenmanni* MLN 0404	0.140771	211741	508521
370461	*Typhlichthys subterraneus* MLN 0296	0.1315116	244871	533541
419871	*Amblyopsis spelaea* YFTC 23868	0.1381242	269351	618871
419871	*Amblyopsis spelaea* YFTC 23869	0.1381242	269351	618871
443681	*Amblyopsis spelaea* YFTC 23875	0.1389843	284171	654811
643881	*Typhlichthys subterraneus* MLN 0051	0.1212618	468701	859611
737491	*Typhlichthys subterraneus* MLN 18	0.1210032	544711	973251
874561	*Typhlichthys subterraneus* UAIC 14148.01	0.1236335	650921	1147671
1136641	*Troglichthys rosae* C3	0.1259494	851821	1484121
1136641	*Troglichthys rosae* C5	0.1259494	851821	1484121
1164901	*Troglichthys rosae* L1	0.1284837	869471	1526761

Table S8. Adaptive Clock Calculation. Calculations of karst cave ages based on generation time estimates for endemic cavefishes.

Node	Median Age (Ma)	Minimum Age (Ma)	Maximum Age	Generation number: Largest per species	Years Since Pseudogenization (15 year gen. time)	Years Since Pseudogenization (5 year gen. time)	Years Since Pseudogenization (3 year gen. time)	Ma Since Pseudogenization (15 year gen time)	Ma Since Pseudogenization (5 year gen time)	Ma Since Pseudogenization (3 year gen time)	Median Ma Since Pseudogenization	Maximum Divergence Time - Max Gen Time	Minimum Divergence Time - Min Gen Time	Median Divergence Time - Med Gen Time	Maximum Divergence Time – 5 Year Gen Time	Minimum Divergence Time – 5 Year Gen Time	Median Divergence Time – 5 Year Gen Time
Troglichthys divergence (tip-dated)	19.8	11.96	29.48	767681	11515215	3838405	2303043	11.515215	3.838405	2.303043	6.909129	17.964785	9.656957	12.890871	25.641595	8.121595	15.961595
Troglichthys divergence (node-dated)	20.35	12.07	30.41	767681	11515215	3838405	2303043	11.515215	3.838405	2.303043	6.909129	18.894785	9.766957	13.440871	26.571595	8.231595	16.511595
Speoplatyrhinus divergence (tip-dated)	11.26	6.02	17.26	167871	2518065	839355	503613	2.518065	0.839355	0.503613	1.510839	14.741935	5.516387	9.749161	16.420645	5.180645	10.420645
Speoplatyrhinus divergence (node-dated)	11.79	6.42	18.42	167871	2518065	839355	503613	2.518065	0.839355	0.503613	1.510839	15.901935	5.916387	10.279161	17.580645	5.580645	10.950645
Amblyopsis spelaea (tip-dated)	1.12	0.11	5.11	286001	4290015	1430005	858003	4.290015	1.430005	0.858003	2.574009	0.819985	-0.748003	-1.454009	3.679995	-1.320005	-0.310005
Amblyopsis spelaea (node-dated)	1.08	0.1	4.7	286001	4290015	1430005	858003	4.290015	1.430005	0.858003	2.574009	0.409985	-0.758003	-1.494009	3.269995	-1.330005	-0.350005
Amblyopsis hoosieri (tip-dated)	1.12	0.11	5.11	204271	3064065	1021355	612813	3.064065	1.021355	0.612813	1.838439	2.045935	-0.502813	-0.718439	4.088645	-0.911355	0.098645
Amblyopsis hoosieri (node-dated)	1.08	0.1	4.7	204271	3064065	1021355	612813	3.064065	1.021355	0.612813	1.838439	1.635935	-0.512813	-0.758439	3.678645	-0.921355	0.058645
Typhlichthys eigenmanni divergence (tip-dated)	7.37	3.38	12	222191	3332865	1110955	666573	3.332865	1.110955	0.666573	1.999719	8.667135	2.713427	5.370281	10.889045	2.269045	6.259045
Typhlichthys eigenmanni divergence (node-dated)	7.83	3.52	13.18	222191	3332865	1110955	666573	3.332865	1.110955	0.666573	1.999719	9.847135	2.853427	5.830281	12.069045	2.409045	6.719045
Typhlichthys subterraneus (tip-dated)	6.17	2.52	10.55	578541	8678115	2892705	1735623	8.678115	2.892705	1.735623	5.206869	1.871885	0.784377	0.963131	7.657295	-0.372705	3.277295
Typhlichthys subterraneus (node-dated)	6.68	2.67	11.63	578541	8678115	2892705	1735623	8.678115	2.892705	1.735623	5.206869	2.951885	0.934377	1.473131	8.737295	-0.222705	3.787295
